# MafB-dependent neurotransmitter signaling promotes β cell migration in the developing pancreas

**DOI:** 10.1242/dev.201009

**Published:** 2023-03-27

**Authors:** Sara Bsharat, Emanuela Monni, Tania Singh, Jenny K. Johansson, Kavya Achanta, Ludivine Bertonnier-Brouty, Anja Schmidt-Christensen, Dan Holmberg, Zaal Kokaia, Rashmi B. Prasad, Isabella Artner

**Affiliations:** ^1^ Lund Stem Cell Center, Lund University, Klinikgatan 26, 22184, Lund, Sweden; ^2^ Lund University Diabetes Center, Jan Waldenströms gata 35, 21428, Malmö, Sweden

**Keywords:** MafB, Pancreas development, Islet formation, Nicotinic acetylcholine receptor, Axon guidance receptor

## Abstract

Hormone secretion from pancreatic islets is essential for glucose homeostasis, and loss or dysfunction of islet cells is a hallmark of type 2 diabetes. Maf transcription factors are crucial for establishing and maintaining adult endocrine cell function. However, during pancreas development, MafB is not only expressed in insulin- and glucagon-producing cells, but also in Neurog3^+^ endocrine progenitor cells, suggesting additional functions in cell differentiation and islet formation. Here, we report that MafB deficiency impairs β cell clustering and islet formation, but also coincides with loss of neurotransmitter and axon guidance receptor gene expression. Moreover, the observed loss of nicotinic receptor gene expression in human and mouse β cells implied that signaling through these receptors contributes to islet cell migration/formation. Inhibition of nicotinic receptor activity resulted in reduced β cell migration towards autonomic nerves and impaired β cell clustering. These findings highlight a novel function of MafB in controlling neuronal-directed signaling events required for islet formation.

## Teaser

**Highlighted Article:** MafB controls expression of neurotransmitter and axon guidance receptor genes that promote islet cell migration and islet formation.

## INTRODUCTION

Pancreatic islets consist predominantly of insulin-producing β and glucagon-producing α cells which are essential for glucose homeostasis. Loss or dysfunction of β cells results in diabetes mellitus. Islet transplantation has proven to be an effective therapy for type 1 diabetes (Triolo and Bellin, 2021). However, donor material is scarce, which makes the production of *in vitro* differentiated islet cells a necessity for cell replacement treatment. Recent reports demonstrated the feasibility of this approach, but increasing evidence suggests that proper glucose control is only achieved if all islet cell types are present and work in concert with one another (Johnston et al., 2016). A thorough understanding of islet development will be required to recreate islet architecture *in vitro* and successfully generate islets for transplantation.

During mouse pancreas development, endocrine progenitor cells initially appear at embryonic day (E) 12.5, but hormone cell production is observed throughout development, with a distinct peak at E15.5. In contrast, postnatal increases in islet cell numbers are caused by proliferation (Bonner-Weir et al., 2016), which decreases in the adult. Mouse islets form by budding of endocrine progenitor cells from pancreatic ducts (Sharon et al., 2019) and aggregation of migrating endocrine cells (Sznurkowska et al., 2020), as signaling cues from the pancreatic mesenchyme (Pauerstein et al., 2017), blood vessels (Staels et al., 2019) and sympathetic neuronal cells (Borden et al., 2013) promote endocrine cell differentiation, with adrenergic signaling from sympathetic nerves promoting β cell expansion and islet formation (Borden et al., 2013).

The MafA and MafB transcription factors are crucial regulators of β cell function and islet immune homeostasis (Artner et al., 2010; Singh et al., 2018; Zhang et al., 2005). MafA also regulates responsiveness of pancreatic islets to autonomic signals in adult islets (Ganic et al., 2016). During pancreas development, the closely related MafB transcription factor is required for α and β cell terminal differentiation, a function that is postnatally taken over by MafA (Artner et al., 2007, 2010; Cyphert et al., 2019; Nishimura et al., 2008). MafB is expressed in a portion of Neurog3^+^ endocrine progenitor cells (Artner et al., 2006), suggesting that MafB may control processes in endocrine cell specification and differentiation before terminal differentiation of endocrine cells.

Here, we show that loss of MafB in developing α and β cells disrupts islet and duct formation. Gene expression analysis revealed that *MafB*-deficient (*MafB^−/−^*) endocrine progenitor cells have reduced expression of several neurotransmitter and axon guidance receptor genes. Single cell sequencing further showed that neurotransmitter and axon guidance receptors were not only expressed in mouse islet β cells, but also co-expressed with MAFB in developing human β cells. In addition, expression of these neurotransmitter receptor genes was reduced in mouse and human *MafB^−/−^* β progenitor cells. Importantly, inhibiting nicotinic acetylcholine receptor (nAchR) function in developing β cells impaired β cell migration and clustering, demonstrating a novel function of nAchRs in islet formation.

## RESULTS

### Loss of MafB in developing endocrine cells disrupts islet and ductal morphogenesis

**Fig. 1.**
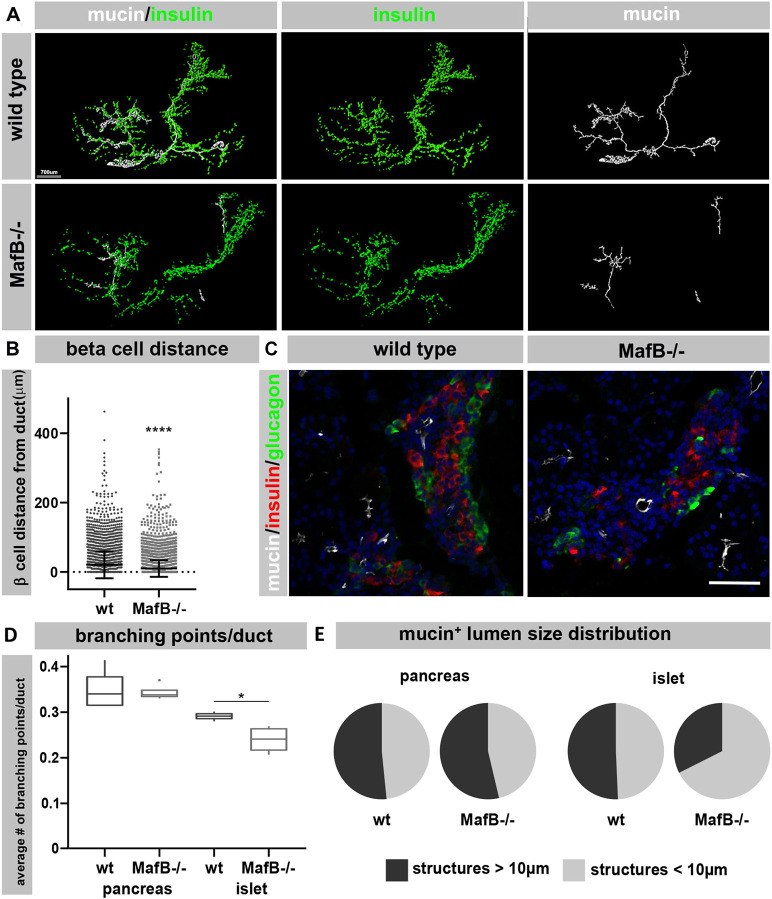
**Whole mount imaging of intact embryonic pancreas.** (A) Embryonic pancreas anlagen from E18.5 wild-type and *MafB^−/−^* pancreata were stained with insulin (green) and mucin (white) and imaged by OPT, surface rendering using Imaris is shown. To visualize larger ductal structures, the ellipsoid prolate filter was applied to the mucin surface tool. The residual ductal structures with major axes shorter than 400 µm were manually removed. Staining of smaller and larger ductal structures is shown in Fig. S1. (B) Distance between insulin^+^ and mucin^+^ surfaces was measured for all islet and ductal structures in wild-type and *MafB^−/−^* pancreata (*n*=4 embryos per genotype from three different litters). ****P<0.0001 (unpaired two-tailed *t*-test). Data are mean±s.e.m. (C) Immunohistochemical staining of wild-type and *MafB^−/−^* E18.5 pancreata with insulin (red), mucin (white), glucagon (green) and nuclei (blue) imaged using a Zeiss LSM780. (D) The average number of branching points in luminal structures of wild-type and *MafB^−/−^* (*n*=4 embryos per genotype) in the pancreatic and islet areas is shown. *P=0.02857 for average branching points of ductal structures in islets (Wilcoxon test). Box plots show a central mark indicating the median and edges indicating 25th and 75th percentiles. Whiskers extend to the largest or smallest point contained within 1.5× of the interquartile range from both edges. (E) Pie charts showing size distribution (>10 µm^2^) of ductal structures in islet and pancreatic exocrine areas. Difference of size distribution of ductal structures between wild-type and *MafB^−/−^* pancreata were significant both in the pancreatic (Chi-square: 165.54; *P*<2.2e^−16^) and islet (Chi-square: 539.49; *P*<2.2e^−16^) areas. *n*=4 embryos per genotype. Scale bars: 700 µm (A); 50 µm (C).

MafB is expressed in α and β cell progenitor and mature α cells in the mouse pancreas. Loss of MafB results in 50% reduction of α and β cell mass at E18.5 (Fig. S1A), while endocrine progenitor cells are present (Artner et al., 2007; Nishimura et al., 2008). To determine whether loss of MafB impairs pancreas/islet morphology, wild-type and *MafB^−/−^* (GFP knock-in; Blanchi et al., 2003) E12.5 and E18.5 pancreata were stained for insulin (β cells) and mucin (ductal cells). 3D optical projection tomography and 2D immunohistochemical analysis showed that wild-type β cells formed large clusters around the developing mucin/ductal network at E18.5 (Fig. 1A) and that *MafB^−/−^* islet cells were located closer to the ductal epithelium (Fig. 1B). Moreover, the organization of the ductal network was impaired in *MafB^−/−^* pancreata, as only few larger ductal structures (Fig. 1A; Fig. S1B) were detected while smaller ductal lumen were present (Fig. S1B, red label), resulting in the total ductal volume not being impaired (Fig. S1C). This was confirmed by immunohistochemical analysis, which showed that the ductal luminal area was not significantly different in *MafB^−/−^* (Fig. S1D), but a significantly larger portion of the ductal structures in contact with islets were small (>10 µm^2^) and the luminal structures had significantly fewer branching points (Fig. 1C-E) than wild type. The average number of ductal branching points did not differ between wild-type and *MafB^−/−^* exocrine tissue (Fig. 1D) and we did not observe morphological changes in ductal volume and organization at earlier time points (E12.5, Fig. S1E), suggesting that loss of MafB in α and β progenitor cells disrupts morphogenesis of islets and associated ductal structures only at later stages of pancreas development.

### MafB is essential for the expression of several neurotransmitter and axon guidance receptor genes in developing β cells

**Fig. 2.**
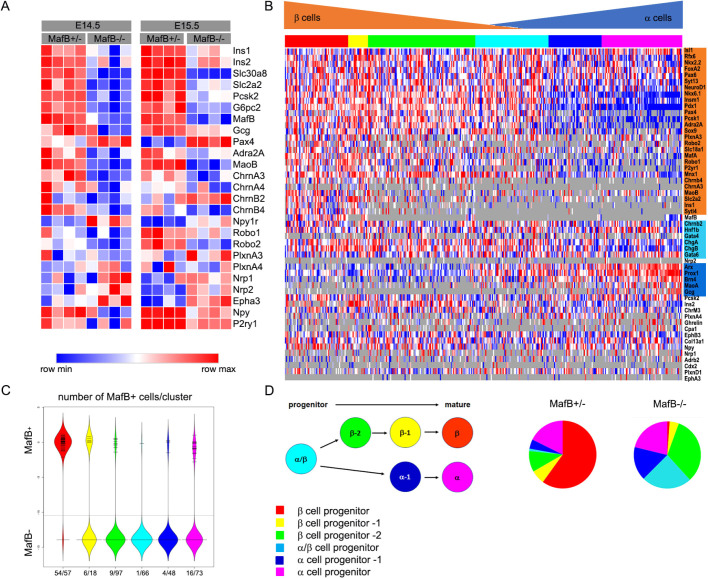
**Gene expression profiles of MafB-GFP^+^ cells in embryonic pancreas.** (A) Heatmap showing global expression profile of *MafB^+/−^* and *MafB^−/−^* E14.5 and E15.5 GFP^+^ cells, *n*=4 biological replicates per genotype: range is 0 (minimum) to 1 (maximum). The heat map was generated using Morpheus web browser from Broad Institute, wherein a relative color scheme uses the minimum and maximum values in each row to convert values to colors. (B) Heatmap showing single cell gene expression of E15.5 *MafB^+/−^* (90 cells) and *MafB^−/−^* (269 cells) GFP^+^ pancreatic cells. Cell clustering using expression profiles was performed using SCExV (Lang et al., 2015) web tool into various stages of α and β progenitor cells. Genes predominantly expressed in α or β progenitor cells are highlighted in blue or orange, respectively, while light blue indicates genes expressed in all endocrine progenitor cells. Range is 0 (minimum) to 1 (maximum). Gray indicates no expression. (C) Number of MafB^+^ cells in the different clusters of α and β progenitor cells. (D) Schematic overview of α and β progenitor cell stages and distribution of *MafB^+/−^* and *MafB^−/−^* cells in these groups using pie charts.

MafB expression is detected in insulin- and glucagon-producing cells as well as a smaller fraction of Neurog3^+^ cells throughout pancreas development (Artner et al., 2006). Loss of MafB expression impairs differentiation of α and β cells in the developing pancreas, while the number of endocrine progenitor cells remains unchanged (Artner et al., 2007). *MafB^+/−^* and *MafB^−/−^* endocrine cells were isolated by fluorescence activated cell sorting (FACS) from E14.5 and E15.5 *MafB* GFP knock-in embryos and global gene expression analysis (cDNA microarray) was performed to identify *MafB* target genes involved in α and β cell differentiation. Wild-type and *MafB^+/−^* pancreata were indistinguishable (Artner et al., 2007), thus *MafB^+/−^* was used to take advantage of the GFP gene knocked into the *MafB* locus (Blanchi et al., 2003). GFP was detected in both heterozygous and *MafB* mutant insulin- and glucagon-producing cells and the number of GFP^+^ cells was comparable between genotypes (2% of all pancreatic cells, Fig. S2). Loss of *MafB* reduced the expression of several known MafB target genes such as *Ins1*, *Ins2*, *Slc30a8*, *Slc2a2*, *Pcsk2* and *G6pc2*, whereas expression of *Pax4*, which marks immature β cells, was increased (Fig. 2A; Table S1). As expected, gene ontology analysis showed that differentially expressed genes were associated with biological processes related to hormone secretion, but also with cell migration and synaptic signaling (Table S2). Interestingly, expression of neurotransmitter receptor genes *ChrnA3*, *ChrnA4*, *ChrnB4*, *P2ry1*, *Adra2A* and the monoamine metabolizing enzyme *MaoB* was significantly reduced at E14.5 and E15.5. In addition, altered expression of axon guidance receptor genes *Robo1*, *Robo2*, *Nrp1*, *Nrp2*, *PlxnA3* and *PlxnA4* was also observed. These data suggest that *MafB* regulates transcription of axon guidance and neurotransmitter signaling pathways which are involved in cell migration and islet formation (Berger et al., 2015; Borden et al., 2013; Pauerstein et al., 2017).

To determine whether expression of genes associated with neurotransmitter signaling were altered in *MafB^−/−^* α and/or β progenitor cells, Fluidigm single cell PCR analysis was performed. Expression analysis of E15.5 *MafB^+/−^* and *MafB^−/−^* GFP^+^ cells showed that the majority of *MafB^+/−^* GFP^+^ cells co-expressed β cell (*Ins1*, *Ins2*, red cell population Fig. 2B,C) or α cell [*Glu* (also known as *Gcg*), *Arx*, *Brn4* (*Pou3f4*), pink cell population] genes. Few cells with lower α or β cell marker expression were detected in the *MafB^+/−^* GFP^+^ cell fraction (yellow, green and blue indicate less mature populations) and only one MafB^+^ cell expressing both α and β cell marker genes (light blue cell population, Fig. 2B,C). In contrast, the majority of *MafB^−/−^* GFP^+^ cells clustered with immature α and β cell progenitor cell populations (yellow, green and blue populations, Fig. 2C). Notably, a large number of cells expressed both α and β cell marker genes (light blue cell population, Fig. 2B). Expression of neurotransmitter and axon guidance receptor (*ChrnA3*, *ChrnB4*, *Robo1*, *Robo2*, *P2ry1*) genes was predominantly observed in *MafB^+/−^* β cells, but reduced/absent in *MafB^−/−^* β progenitor cells, whereas none of our newly identified MafB target genes was specifically expressed in α cells. The majority of the *MafB^+/−^* cells at E14.5 and E15.5 were β cell progenitors [roughly 75% (60/90 MafB^+^ cells), Fig. 2D] resulting in the identification of MafB target genes predominantly in β cells at these time points.

### Neurotransmitter and axon guidance gene expression is maintained in maturing α and β cells

**Fig. 3.**
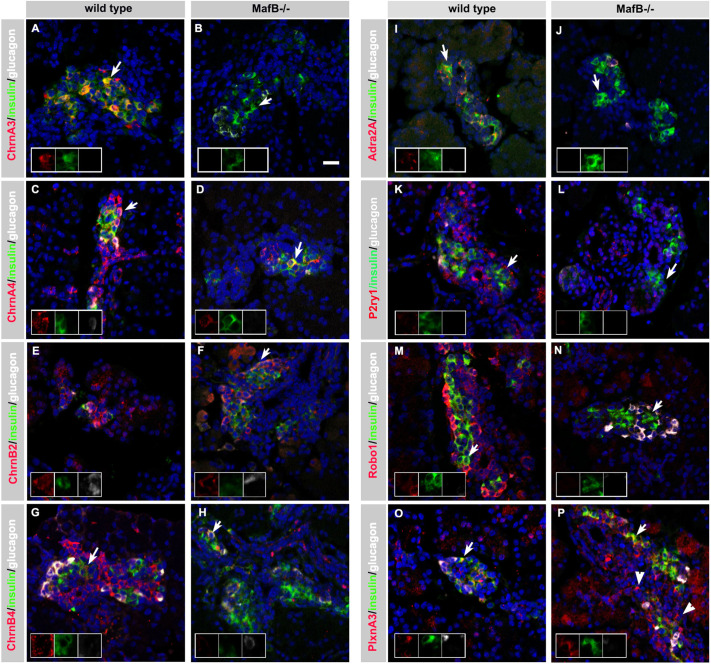
**Neurotransmitter receptor expression in embryonic pancreas.** (A-P) Immunohistochemical analysis of neurotransmitter receptor expression in wild-type and *MafB^−/−^* E18.5 pancreas. Co-staining for insulin (green) and glucagon (white) with neurotransmitter receptor proteins in red – ChrnA3 (A,B), ChrnA4 (C,D), ChrnB2 (E,F), ChrnB4 (G,H), Adra2A (I,J), P2ry1 (K,L), Robo1 (M,N) and PlxnA3 (O,P) – visualized using a Zeiss LSM780. Nuclei are stained in blue. Arrows mark insulin producing cells co-expressing respective neurotransmitter receptor proteins, arrowheads denote PlxnA3^+^ cells in (P), insets show co-expression of neurotransmitter receptor proteins with insulin and glucagon. Scale bar: 20 µm.

Immunohistochemical analysis of E18.5 wild-type and *MafB^−/−^* pancreata was performed to determine whether neurotransmitter and axon guidance receptor expression is maintained throughout islet differentiation and dependent on the presence of MafB in maturating α and β cells. ChrnA3, ChrnA4, ChrnB4, P2ry1 and Robo1 were detected in developing wild-type α and β cells, as well as adjacent epithelial cells (Fig. 3) and expression was reduced in *MafB^−/−^* pancreata, with no (ChrnA3, P2ry1; Fig. 3B,L) and only few (ChrnA4, ChrnB4, Robo1; Fig. 3D,H,N) neurotransmitter receptor-expressing cells remaining. Adra2A expression was exclusively detected in the membrane of wild-type β cells, and was lost in *MafB^−/−^* pancreata (Fig. 3I,J). In contrast, ChrnB2 expression was maintained in *MafB^−/−^* cells (Fig. 3E,F) and PlxnA3, which is expressed in wild-type α and β cells, was detected in non-hormone producing mutant cells (Fig. 3O,P). The protein expression analysis largely confirmed our results obtained using single cell PCR (Fig. 2B) and demonstrates that neurotransmitter and axon guidance receptor expression is impacted upon MafB loss during late pancreas development.

### Neurotransmitter and axon guidance genes are expressed in fetal pancreata and associated with type 2 diabetes development

**Fig. 4.**
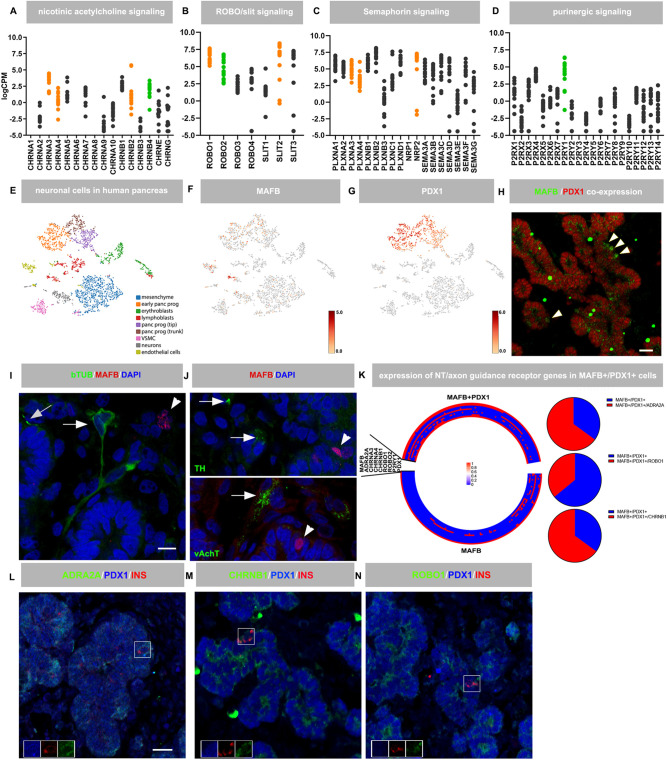
**Neurotransmitter and axon guidance signaling in human embryonic pancreas.** (A-D) RNA-seq of human embryonic pancreas (7-14 weeks gestation) of nicotinic (A), Robo/Slit (B), semaphorin (C) and purinergic (D) signaling (*n*=16). Genes that were differentially expressed in mouse *MafB*^−/−^ GFP^+^ cells are indicated in orange, genes with impaired expression in *MafB*^−/−^ GFP^+^ cells and reported type 2 diabetes risk alleles are indicated in green. Data were expressed as log counts per million mapped reads (logCPM), *n*=16. (E) t-SNE visualization of single cell sequencing data from human embryonic pancreas (Carnegie stage 22, *n*=3199). Clusters were identified using top 20 expressed genes per cluster (Table S3). Neural cells are labeled in gray. (F,G) t-SNE showing expression of MAFB (F) and PDX1 (G). (H-J) Co-staining of PDX1 (red) and MAFB (green), arrows depict cells co-expressing PDX1 and MAFB (H), β III-tubulin (I), and vAchT and TH (J) with MAFB in human embryonic pancreas (Carnegie stage 22). Arrows depict nerve fibers, arrowheads show MAFB^+^ nuclei (I,J). (K) Co-expression analysis (circular plot) of *MAFB^+^* and *MAFB*^+^/*PDX1^+^* cells with *ADRA2A*, *CHRNA3*, *CHRNA4*, *CHRNB1*, *ROBO1*, *ROBO2* and *P2RY1* in human fetal scRNA-seq, pie-charts illustrating co-expression of *ADRA2A*, *CHRNB1*, and *ROBO1* with *MAFB* and *PDX1*. (L-N) Co-staining of ADRA2A (L, green), CHRNB1 (M, green), and ROBO1 (N, green) with insulin (white) and PDX1 (red). Nuclei are in blue. Insets show expression of insulin (red), PDX1 (blue) and the respective receptor (green) in single channels. Scale bars: 10 µm (I,J); 20 µm (H,L-N).

To evaluate whether neurotransmitter and axon guidance signaling genes are also expressed in the developing human pancreas, gene expression analysis of human pancreas anlagen was performed. Bulk RNA-seq of 7-14 weeks post conception (pc) pancreatic tissue (*n*=16) showed that neurotransmitter receptor and axon guidance genes are expressed in human pancreas anlagen (Fig. 4A-D). Single cell sequencing (scRNA-seq) and immunohistochemical analysis of human embryonic pancreas (Carnegie stage 22, 3199 cells) showed the presence of endocrine progenitor cells (MAFB^+^/PDX1^+^, Fig. 4F-H) and a distinct cluster of autonomic nerve cells (Fig. 4E,I,J; Fig. S3). Sympathetic and parasympathetic nerve cells were detected close to human and mouse MAFB^+^ cells (Fig. 4I,J; Fig. S4A). PDX1, MAFB, ADRA2A, CHRNB1 and ROBO1 were expressed in pancreatic progenitor cell clusters (Fig. 4F,G, Fig. S3) and co-expression analysis of MAFB and PDX1 with neurotransmitter and axon guidance receptor genes showed that a significant portion of *MAFB^+^*/*PDX1^+^* β progenitor cells expressed adrenergic, axon guidance and nicotinic receptor genes (Fig. 4K; Fig. S3). Several *MAFB^+^*/*PDX1^+^* cells expressing multiple neurotransmitter/axon guidance receptor genes (Fig. 4K) were detected. Expression of CHRNB1, ADRA2A and ROBO1 in the pancreatic epithelium, MAFB- and insulin-producing cells was also detected using immunohistochemical staining of human embryonic pancreas (Fig. 4L-N; Fig. S4B).

To evaluate whether loss of MAFB also affects expression of adrenergic, nicotinic and axon guidance receptor genes, scRNA-seq of *in vitro* differentiated β cells derived from MAFB-deficient human embryonic stem cells and MAFB ChIP-seq from ENDOCβH2 β cells (Russell et al., 2020) were re-analyzed. Expression of *CHRNA3*, *CHRNB2*, *ROBO2*, *NRP1*, *NRP2* and *ADRA2A* was reduced in MAFB-deficient β-like cells (Table 1). In addition, MAFB binding to *CHRNB2*, *ROBO1*, *ROBO2* and *NRP1* genomic regions was detected in the human embryonic ENDOCβH2 β cells (Table 1), suggesting that MAFB directly binds to and activates these genes during human β cell development. Interestingly, expression levels of several of these genes were also correlated with *MAFB* expression levels in adult islets (Table 1), MAFB binding to neurotransmitter (*P2RY1*) and axon guidance receptor (*ROBO1*, *ROBO2*, *NRP1*, *NRP2*) genes was also detected in adult human islets (Table 1) and quantitative trait loci (eQTL) influencing islet gene expression (Fig. S5; Table 1; *PLXNA4*, *ROBO2*, *P2RY1*) were identified. We also identified *P2RY1*, *ROBO2* and *PLXNA4* variants which were nominally associated with gene expression levels of their respective genes and also showed suggestive signals of association with insulin secretion indices (Fig. S4; Table S3; Prokopenko et al., 2014; Wood et al., 2017). These data indicate that transcriptional regulation of several neurotransmitter and axon guidance receptor genes depends on MAFB expression, is conserved between human and mouse, and is relevant to adult islet function.

**Table 1.**
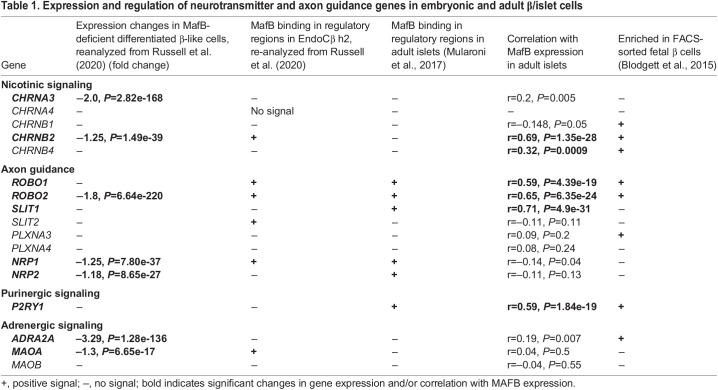
Expression and regulation of neurotransmitter and axon guidance genes in embryonic and adult β/islet cells

### nAchR signaling is required for β cell-nerve cell interactions

**Fig. 5.**
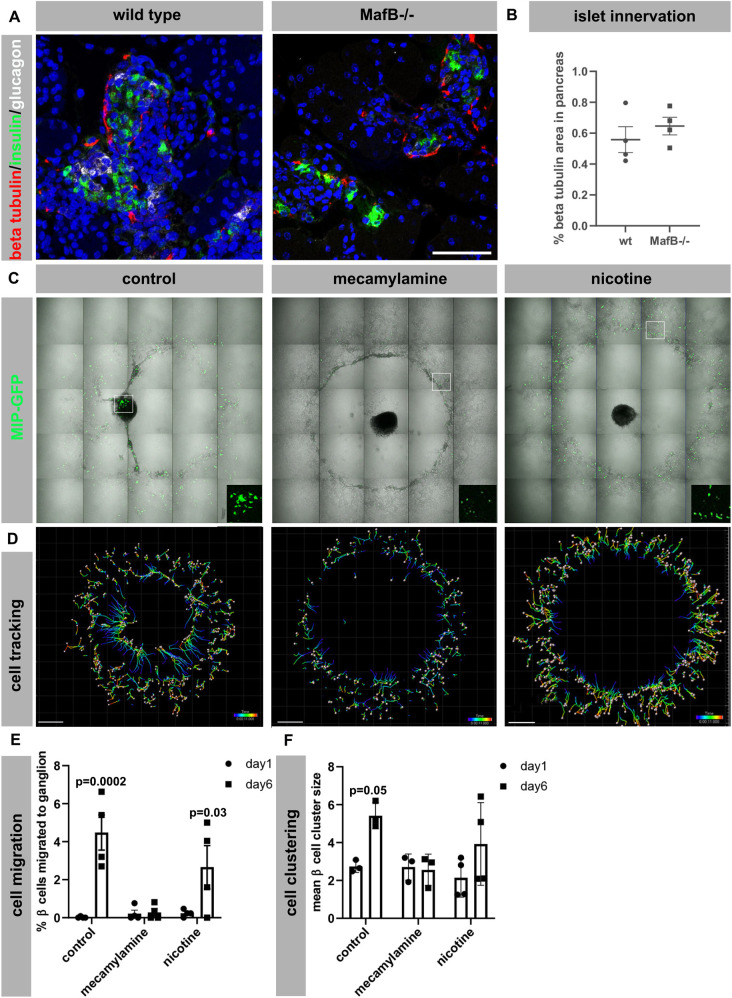
**Nicotinic receptor function in embryonic islet development.** (A) Immunohistochemical staining of wild-type and *MafB^−/−^* E18.5 pancreas for insulin (green), β III-tubulin (red) and glucagon (white). Nuclei are in blue. (B) β III-tubulin^+^ area in E18.5 wild-type and *MafB^−/−^* pancreas. *n*=4 per genotype. Error bars indicate s.e.m. (C) Tile scan of co-cultures of MIP-GFP^+^ (green) β cells and superior cervical ganglia (black, placed in the center) treated with mecamylamine, nicotine, and control for 6 days imaged with a Zeiss LSM780 confocal microscope, objective 5×. Insets show GFP expression. (D) Visualization of cell tracking of MIP-GFP^+^ β cells during 6 days of co-culture using Imaris software (version 9.6.0). (E) Quantification of MIP-GFP^+^ cells located in ganglion, *n*=3-4 per condition. (F) Mean MIP-GFP^+^ cluster size after 6 days of co-culture, *n*=3-4, error bars indicate s.d. Significance of changes between day 1 and 6 for each condition was analyzed using two-way Anova with Sidak's multiple comparison test (E and F). Scale bars: 50 µm (A); 700 µm (C,D).

Our analysis has shown that loss of MAFB impairs expression of several neurotransmitter (*ChrnA3*, *ChrnA4*, *ChrnB4*, *Adra2A*, *P2ry1*) and axon guidance receptor (*Robo1*, *Robo2*) genes in mouse and human β cells. To evaluate whether loss of MafB and concomitant changes in gene expression altered islet innervation, immunohistochemical stainings of insulin and glucagon, with the neuronal marker β III tubulin and the glial marker Sox10 were performed at E18.5 in mouse wild-type and *MafB^−/−^* pancreata. β III tubulin^+^ nerve fibers and Sox10^+^ glial cells were readily detected in the developing pancreas and surrounding tissues (Fig. 5A). The β III tubulin^+^ cell area within the pancreas and pancreatic ganglia and Sox10^+^ area in pancreatic ganglia were not altered between wild-type and *MafB^−/−^* pancreata indicating that loss of MafB is not sufficient to impair islet innervation of the mutant pancreata (Fig. 5B; Fig. S4C), possibly due to continued expression of NGF and GDNF (Table S1) which are crucial for islet innervation (Glebova and Ginty, 2005).

Previous studies have demonstrated that adrenergic signaling is required for β cell migration towards developing sympathetic nerves (Borden et al., 2013). To evaluate whether loss of nAchR activity also impaired β cell migration towards autonomic nerves, we examined how migration was impacted upon co-culturing developing β cells from mouse insulin promoter GFP transgenic animals (MIP-GFP^+^; Hara et al., 2003) with superior cervical ganglia (acetylcholine source, Fig. S4D) in the presence of nicotinic receptor agonists (nicotine) or antagonists (mecamylamine). Untreated MIP-GFP^+^ β cells migrated towards the centrally located ganglion (Fig. 5C-E) and 5% of MIP-GFP^+^ cells were located within the ganglionic structure after 6 days of co-culture. In contrast, blocking nicotinic signaling with mecamylamine resulted in a loss of directed migratory behavior with no accumulation of MIP-GFP^+^ cells in the ganglionic structure (Fig. 5C-E). Treatment of co-cultures with nicotine, which activates nAchRs, also did not result in a significant accumulation of MIP-GFP^+^ cells in ganglionic tissue, most likely due to loss of directionality (no gradient formed) and/or receptor desensitization. Moreover, co-culture of ganglionic tissue and MIP-GFP^+^ cells promoted accumulation of β cells in clusters (Fig. 5C,F). MIP-GFP^+^ cell clustering was impaired upon treatment with mecamylamine (Fig. 5F), whereas nicotine treatment affected clustering to a lesser extent. These data suggest that nAchR activity (which is impaired in *MafB^−/−^* β cells) is required for β cell clustering and migration towards autonomic tissue.

## DISCUSSION

Maf transcription factors contribute to β cell development and adult β cell function. Previous studies have shown that loss of MafB impairs α and β cell terminal differentiation during late embryonic pancreas development (Artner et al., 2007; Nishimura et al., 2008). Here, we examined expression profiles of MafB-deficient endocrine progenitor cells at E14.5 and E15.5 to identify MafB target genes controlling α and β cell commitment during the secondary transition of β cell development. Analysis of MafB mutant embryonic pancreata demonstrate that MafB promotes the rapid progression of α and β cell progenitors to mature endocrine cells and subsequently islet formation. MafB mutant β cells had altered expression of axon guidance (*Robo1*, *Robo2*, *Nrp1*, *Nrp2*) and neurotransmitter receptor (*ChrnA3*, *ChrnA4*, *ChrnB4*, *P2ry1*, *Adra2A*) genes, suggesting that MafB controls responsiveness to environmental cues, which may contribute to endocrine cell differentiation, migration and islet formation during pancreas development.

Maf transcription factors MafA and MafB are crucial for adult β cell function by controlling transcription of glucose sensing, insulin production and secretion genes. MafB activates Pdx1 and MafA transcription in developing β cells, thereby promoting terminal differentiation of β cells (Artner et al., 2007). MafB expression in Neurog3^+^ progenitor cells suggests additional functions in α and β cell commitment. Our single cell gene expression analysis of endocrine progenitor cells showed that most MafB^+^ endocrine progenitor cells expressed mature α or β cell marker genes. In contrast, the vast majority of *MafB^−/−^* cells had a more immature expression profile and a significant proportion of cells expressed both α and β cell marker genes (e.g. *Pdx1*, *Pax4* and *Arx*), suggesting that lack of MafB inhibits, or at least delays, specification of α and β cells. This notion was further supported by previous studies illustrating that selective deletion of MafB using Pdx1-Cre resulted in impaired β cell differentiation that was eventually compensated for by adult MafA expression (Conrad et al., 2016). Non-committed α/β cell progenitor cells were also detected in the MafB^+^ endocrine progenitor population, albeit at very low numbers, indicating that progenitor cells quickly transition through this stage upon initiation of MafB expression (Fig. 2D). A similar accumulation of immature endocrine progenitor cells was also observed in β-like cells derived from MAFB-deficient human embryonic stem cells (ES cells) (Russell et al., 2020), but loss of MAFB in this *in vitro* setting also resulted in an enrichment of somatostatin-producing delta cells, which we did not observed in mouse pancreas anlagen. This may reflect species differences in MafB function or may be due to differences in cell culture versus *in situ* development of the pancreas.

We re-evaluated whether loss of MafB impaired islet formation and pancreas morphogenesis. Interestingly, whole mount image analysis showed that E18.5 *MafB^−/−^* pancreata had defects in the formation of larger ductal structures and that the remaining β cells were closely associated with smaller ducts. This was somewhat surprising considering that E12.5 *MafB*^−/−^ pancreas anlagen were indistinguishable from wild-type littermates and that MafB expression was only detected in the endocrine lineage from E10.5 (Artner et al., 2006; Nishimura et al., 2006), with no expression in ductal cells been reported so far. During pancreas development, endocrine progenitor cells exit the pancreatic epithelium to form islets of Langerhans and transcription factors important for these processes have been identified (Bankaitis et al., 2018). For example, epithelial malformations including impaired ductal branching were also observed in tissue specific knockout models of *Hnf1b* (De Vas et al., 2015), *Hnf6* (*Onecut1*; Pierreux et al., 2006), *Neurog3* (Magenheim et al., 2011) and *Prox1* (Westmoreland et al., 2012). Interestingly, the defects in ductal branching were only observed in the vicinity of mutant islets, suggesting that the morphogenetic changes observed in *MafB^−/−^* ductal organization may arise from defects in α and β cell migration/exit from the epithelium.

Endocrine cell migration from the pancreatic epithelium and subsequent islet formation depend on external cues from the mesenchyme, blood vessels and autonomic nerves. Gene expression profiling of *MafB^−/−^* endocrine progenitor cells showed that expression of several axon guidance and neurotransmitter receptor genes was altered in *MafB*^−/−^ β progenitor cells. Previous studies by Pauerstein et al. have implicated semaphorin signaling from the mesenchyme in the initiation of α and β cell migration/islet formation (Pauerstein et al., 2017) and show that Nrp/Plxn receptors are expressed in α and β progenitor cells. Consistent with this finding, we detected an upregulation of Nrp and Plxn receptors in *MafB^−/−^* endocrine progenitor cells. In contrast, *Robo1* and *Robo2* axon guidance receptor expression was reduced in *MafB* mutant endocrine cells, which may contribute to the defects in islet formation observed in the knockout as Robo/Slit signaling participates in islet cell sorting and morphogenesis (Adams et al., 2018).

MafB mutant β cells had drastically reduced expression of adrenergic, nicotinic and purinergic receptor genes (*Adra2a*, *ChrnA3*, *ChrnA4*, *ChrnB4*, *P2ry1*). Acetylcholine and epinephrine secretion has been reported from autonomic nerves and blood circulation, but also α cells (acetylcholine; Rodriguez-Diaz et al., 2011b) in the islet, where epinephrine and acetylcholine regulate insulin secretion in the adult islet via nicotinic, muscarinic and adrenergic receptors expressed in β cells (reviewed by Rorsman and Ashcroft, 2018). During development, adrenergic signaling from pancreatic nerves is crucial for islet formation, as blocking of β adrenergic receptors reduces β cell numbers and impairs migration of β cells to autonomic nerves (Borden et al., 2013). We show that β cell migration and clustering is impaired upon inhibition of nicotinic signaling in co-cultures of embryonic β cells with autonomic nerves, suggesting that acetylcholine signaling contributes to islet innervation and formation. A similar effect was observed upon addition of nicotine to co-cultures, indicating that a gradual activation of receptors is required for providing a directional cue that results in migration of β cells to pancreatic nerves while uniform exposure does not allow for directed migration. Previous studies have shown that nicotinic receptors participate in controlling migratory behavior of bronchial epithelial cells (Tournier et al., 2006) by modulating intracellular Ca^2+^ levels, suggesting that a similar mechanism may be in place in developing β cells.

Differences in adult islet innervation suggest that parasympathetic acetylcholine signaling contributes to adult β cell function in rodents, but not in humans (Rodriguez-Diaz et al., 2011a). However, immunohistochemical studies in fetal human pancreas show interactions between nerve and islet cells (Krivova et al., 2016) and we have detected a distinct nerve cell population in our scRNA-seq (Fig. 4E, gray) and immunohistochemical (Fig. 4I,J; Fig. S4B) analysis of human embryonic pancreas, suggesting that autonomic nerves may also play a role in human islet morphogenesis. Transcripts and protein of neurotransmitter receptor genes were readily detectable in human embryonic pancreas anlagen and developing β cells further supporting a role of these signaling pathways in human pancreas development. The relationship between *MAFB* and neurotransmitter receptor expression is conserved between differentiating human and mouse β cells as human *MAFB^+^/PDX1^+^* cells co-express *ADRA2A*, *CHRNB1* and *ROBO1,* and nicotinic, adrenergic and axon guidance receptor gene expression was impaired in MAFB-deficient human β-like cells. Moreover, correlation of MAFB expression with expression of several neurotransmitter receptor and axon guidance genes in adult human islets indicates that these signaling pathways have a dual role during islet development and in adult islet function. This notion is further supported by the presence of type 2 diabetes susceptibility signals intersecting eQTLs for the *ADRA2A* (Rosengren et al., 2010), *CHRNB4* (Ganic et al., 2016) and P2RY1 genes.

Here, we describe a novel function of the MafB transcription factor in islet morphogenesis by controlling neurotransmitter sensing in developing β cells. Specifically, we identify nicotinic signaling as a key mechanism in islet-nerve cell communication and β cell migration and clustering. These findings describe a novel mechanism by which prenatal nicotine exposure, which has been associated with an increased risk of type 2 diabetes, development and impaired endocrine cell development (Somm et al., 2008), may affect islet formation and subsequent β cell function.

## MATERIALS AND METHODS

### Animals

MafB-GFP knock-in (*MafB^−/−^*) (Blanchi et al., 2003) and MIP-GFP (Hara et al., 2003) mouse lines have been previously described. Mice were maintained on a C57BL/6 background. Experimental procedures were performed using embryonic pancreata (E14.5, E15.5 and E18.5). All animal procedures were pre-approved and performed according to Swedish national guidelines (approval number 5733/2018).

### Immunohistochemistry/whole mount staining

For immunostaining, pancreata from E18.5 wild-type and *MafB^−/−^* embryos and human embryonic pancreas (Carnegie stage 22) were dissected, fixed in 4% paraformaldehyde (Sigma-Aldrich) and embedded in paraffin (Thermo Fisher Scientific) before being sectioned (6 µm). Immunohistochemical analysis was performed as previously described (Matsuoka et al., 2003). The primary antibodies used were: guinea pig α-insulin (1:2000, Dako, A0546), mouse α-glucagon (1:2000, Sigma-Aldrich, G2654), rabbit α-CHRN-A3/A4/B2/B4 (ANC-003, ANC-004, ANC-012, ANC-014, respectively; 1:100; Alomone Labs), rabbit α-CHRNB1 (1:100, Abcam, AB 126234), rabbit α-Adra2A, (1:100, Sigma-Aldrich, A271), goat α-Adra2A (1:100 Sigma-Aldrich, SAB2500033), mouse α-Robo1 (v-Robo1-s, 1:100, DSHB, 13C9), rabbit α-PlxnA3 (1:100, Alomone Labs, APR-093), rabbit α-P2RY1 (1:100, Alomone Labs, APR-009), rabbit α-GFP (1:200, Molecular Probes, A11122), Armenian hamster α-mucin (1:500, Thermo Fisher Scientific, 1630-R7), goat α-PDX1 (1:1000, Abcam, ab47383), rabbit α-MAFB (1:250, Atlas Antibodies, HPA005653), mouse α-MAFB (1:50, Bio-Techne, NBP2-45718), goat α-Sox10 (1:200, Santa Cruz, Sc-17343), rabbit α-β-tubulin (1:5000; Covance, PRB-435P), mouse α-vAchT (1:100, Thermo Fisher Scientific, MA5-27662), sheep α-Tyrosine Hydroxylase (1:200, Thermo Fisher Scientific, PA1-4679). Secondary antibodies used were Cy2-, Cy3- and Cy5-conjugated α-guinea pig, α-mouse, α-Armenian hamster, α-goat and α-rabbit (1:500; Jackson ImmunoResearch, 706-225-148, 706-165-148, 706-175-148, 715-225-151, 715-165-151, 715-175-150, 127-225-160, 705-225-147, 705-165-147, 705-175-147, 711-225-152, 711-165-152 and 711-175-152). Nuclear counterstaining was performed using DAPI (1:6000; Thermo Fisher Scientific). Neurotransmitter/axon guidance receptor antibodies were previously validated in the following publications: Adra2A, ChrnA4, ChrnB2, ChrnB4 (Ganic et al., 2016), P2ry1 (Caputi et al., 2017), ChrnB1 (proteinatlas.org) and Robo1 (Glawe et al., 2013).

### Histological analysis of embryonic pancreas

E18.5 wild-type and *MafB^−/−^* pancreata (*n*=4 in each group) were paraffin embedded and sectioned (see above). For histological analysis, sections with a distance of 70 µm were stained for Sox10, β III tubulin or mucin with insulin, glucagon and DAPI. Stained sections were scanned on an Olympus slide scanner (VS120) with a 20× objective. Pictures were analyzed using Pathology AI (Visiopharm). The application was first trained to recognize islet, pancreatic, gut and ganglionic structures from wild-type and *MafB^−/−^* sections by manual outlining of the respective structures, the application was then allowed to train for 143,000 iterations using Deep Labv3^+^ classification. Accuracy of tissue recognition was assessed afterwards. Insulin-, glucagon-, β III tubulin-, mucin- and Sox10-stained area was then determined using a fluorescence threshold, and respective areas were then normalized against pancreatic, islet or ganglionic areas. Mucin structures were categorized into stained areas larger or smaller than 10 µm^2^ and the size composition of mucin lumina per islet and pancreatic area was assessed. Branching points were automatically recognized and the average number of branching points per mucin structure in islet versus pancreatic area determined.

### Optical projection tomography (OPT)

OPT scans were performed using the Bioptonics 3001 OPT M scanner (Bioptonics) with exciter D560/40× and emitter E610lpv2 filter (Chroma). Tomographic re-constructions were generated using the NRecon V1.6.1.0 (Skyscan) software, and reconstructed images were further assessed using the Bioptonics viewer V2.0 as previously described (Alanentalo et al., 2010). Final images were made by using Imaris (version 9.6.0, Oxford Instruments).

### Imaris image analysis

Fluorescence images datasets obtained from OPT scans of individual pancreata were converted and analyzed using the Imaris software (version 9.6.0 and 9.9.1, Oxford Instruments). The stomach had to be removed from each dataset due to its strong positivity for mucin. In order to do this, the contour tool was used and an ad hoc surface object for the stomach was created manually. Subsequently the newly created surface was used as a Channel Mask and the voxels inside the surface were set to zero in order for the surface to be deleted. Insulin-positive cells and mucin-positive ducts were segmented using the ‘Surface’ object tool. To obtain the distance measurement between β cells and ducts, ‘Distance Transformation’ within the XTension tool was used. The distance was calculated outside of the β cells and any ductal lumen surface. The ‘Surface’ object tool was used to get measurements of the ductal volume.

Time-lapse movies of *in vitro* islet migration assays obtained using a laser scanning confocal microscope (Zeiss 780) were analyzed with the Imaris software (version 9.6.0). GFP^+^ β cells were identified and tracked over time using the ‘Spots’ function. Individual cells are visualized as green spheres (Spot Objects) with a radius set to 5 µm, and tracks are displayed as time-color coded Full Tracks. The maximum distance that a tracked Spot could move between two consecutive time points was set to 180 µm.

### Isolation of GFP^+^ murine pancreatic cells for global gene expression analysis

E14.5 and E15.5 *MafB* GFP knock-in pancreata (*MafB^+/−^* and *MafB^−/−^*) were dissected in cold PBS and dissociated with Accumax solution (Sigma-Aldrich) containing 5 μg/ml DNase I (Sigma-Aldrich) at 37°C for 1 h with periodic resuspension. Quenching solution [L15 medium, 10 mM Hepes (pH 7.0), 1 mg/ml bovine serum albumin (Thermo Fisher Scientific), 5 μg/ml DNase I] was added to stop the reaction. The cell suspension was filtered through a 30 μm filter into FACS tubes, washed with quenching solution and resuspended in 0.5 ml quenching solution containing 7AAD viability dye (1:2000; Thermo Fisher Scientific). Single GFP^+^ cells were sorted directly into Trizol LS reagent (Thermo Fisher Scientific) using a FACS Aria II cell sorter (BD Biosciences). RNA extracted from 2000-5000 GFP^+^ cells was labeled, hybridized to the Mouse Gene 2.0/2.1 Array (Affimetrix) and scanned according to the manufacturer's instructions.

Scanned image files were first inspected for quality control (QC) using built-in QC tools from the Bioconductor package of R. QC consisted of visual examination of probe array images, scatter plots from replicates, hierarchical clustering of array hybridizations, RNA degradation plots and MvA plots. Detection calls indicating the presence or absence of signal from each probe set were obtained by processing the raw data with the Microarray Analysis Suite 5.0 (MAS5). Feature intensity values from scanned arrays were normalized and reduced to expression summaries using the Robust Multiarray Algorithm (RMA) implemented in the affy package in R (Gautier et al., 2004) and log2 counts were obtained.

Probesets 3′ locations were obtained by downloading the respective probe tab files made available by the Affymetrix online support. A probeset location was considered equal to the 3′ distance of the probe that is most distal from the 3′ end of the corresponding target within the set.

To test for differential expression, the Bayesian adjusted *t*-statistics from the linear models for Micoarray data (limma) package was implemented (Ritchie et al., 2015). A multiple testing correction based on the false discovery rate (FDR) was performed and FDR corrected *P*-values of <0.05 were considered to be significant.

### Gene expression analysis using Biomark Fluidigm dynamic arrays

E15.5 single GFP^+^ cells from *MafB^+/−^ and MafB^−/−^* pancreata were sorted using FACS ARIA II cell sorter (BD Biosciences) directly into a 96-well plate containing lysis buffer. A detailed procedure using Biomark 96.96 dynamic array platform (Fluidigm) and gene expression analysis has been previously described (Singh et al., 2019). Primer sequences are presented in Table S4 and Larsen et al. (2017). Data were further analyzed and heatmaps/violin plots were generated with SCExV using hierarchical clustering (Lang et al., 2015).

### Human tissue

#### Human fetal pancreas

Human fetal pancreas tissue was obtained from material available following elective termination of pregnancy at the University Hospital in Malmö, Sweden. The study was approved by the national ethics committee (permit number 2018-579), written and oral consent was obtained before collection. Fetal/embryonic age was determined by measuring crown-rump length and Carnegie stage, where appropriate. Pancreata were either dissected and homogenized in Trizol and further extracted using RNeasy Mini columns (Qiagen) for bulk RNA extraction (Mazur et al., 2013) or single cell suspensions were prepared using a combination of dissociation enzymes accutase (Sigma-Aldrich) and liberase (Roche) (in 10:1 dilution, respectively). Fetal pancreas/enzyme mix was incubated at 37°C for 30 min with continuous shaking and periodic resuspension at 10-min intervals. Single cells were immediately spun at 1100 rpm (100 ***g***) at 4°C for 5 min and resuspended in cold DPBS Ca^2+/^Mg^2+^ free buffer (Thermo Fisher Scientific) with 5% fetal bovine serum (Sigma-Aldrich), 0.5 mM EDTA (Sigma-Aldrich) and 5 μg/ml DNase I (Sigma-Aldrich). After washing, the cell suspension was filtered through a 30 μm filter (BD Biosciences), viability was assessed, and 8000 cells were resuspended and loaded into a 10x Genomics Chromium Single Cell System.

#### Single cell sequencing and analysis

Libraries were generated using version 3 chemistry according to the manufacturer's instructions (10x Genomics). Libraries were sequenced on Illumina NextSeq500 (400 million reads flow cells) using the recommended read length with 42 bp paired-end reads. Sequencing data were processed through the Cell Ranger pipeline (10x Genomics, Cell Ranger version 6.0.1) to perform demultiplexing using the ‘mkfastq’ function. Resulting sequences were aligned to GrCH38 (v3.1.0), filtered using default parameters, and barcode and UMI counting was performed using the CellRanger ‘count’ function. In total, 3199 cells were obtained and included in the analysis. Unsupervised graph-based clustering was performed using Loupe browser and validated using custom R scripts. Graph-based clustering method is facilitated by building a graph where each node is connected to its nearest neighbors in high-dimensional space, with edges being weighted based on similarity between closely related cells. Assumption-free clustering of cells is then performed based on this similarity. Clusters were assigned using top 20 expressed genes (Table S3).

Single cell sequencing data from human *MAFB*^+/+^ and *MAFB* ^−/−^ β-like cells (derived from iPSCs) were re-analyzed and expression of neurotransmitter receptor genes determined (Russell et al., 2020). The matrix of gene counts versus cells from this study was re-analyzed. The R package Seurat (version 2.3.4) was used to process the unique molecular identifier count matrix (Satija et al., 2015) and to perform data normalization (gene expression measurements for each cell were normalized by total expression and log-transformed), dimensionality reduction and clustering, and the Wilcoxon rank sum test was used for differential expression (DE) analysis.

#### Bulk RNA-seq and analysis

Sequencing data were demultiplexed using bcl2fastq. The resulting sequences were aligned against the human genome build 38 using STAR v2.4.1 and gene features were counted using ‘featurecounts v 1.4.4’ (*Liao et al., 2014*), with GENCODE version 22. Raw counts were normalized using trimmed mean of M-values and log_2_-transformed. Spearman correlations (R) were calculated to assess correlation of *MAFA* and *MAFB* expression with genes expressed in the human pancreatic islets using R language programming. eQTL analysis was carried out as previously described (Asplund et al., 2020; Fadista et al., 2014) using islets from the same 191 organ donors using the R Matrix eQTL packages (Shabalin, 2012). eQTLs for genes of interest were extracted and looked-up further for association with type 2 diabetes and insulin secretion in previous GWAS datasets, from DIAGRAM, DIAMANTE and MAGIC datasets.

#### Statistical analyses

All graphs and statistical analyses were carried out using GraphPad Prism software. Unless otherwise noted in the figure legends, statistical significance was determined using an unpaired two-tailed Student's *t*-test. Details regarding replicates are indicated in the respective figure legends.

#### *In vitro* islet migration assays

Co-cultures were performed as previously described (Borden et al., 2013) with the following modifications: Cultures were maintained for 6 days at 37°C in 5% CO_2_, with 10% fresh medium/H_2_O solution (1:1) added daily. In experiments using nicotinic antagonists, after plating, cultures recovered overnight in culture media containing NGF alone. The next day, fresh medium and mecamylamine (Sigma-Aldrich) or nicotine (Sigma-Aldrich) were added. NGF was added every other day. Cultures were plated in duplicates for each condition.

Cultures were imaged every 10 h, with time point one being the day after plating, using a Zeiss LSM780 microscope and a 10× objective. *Z*-stacks were taken with 20 µm between images and 25 tiles to cover the whole area, images were stitched and maximum projection generated for subsequent analysis (Zen software version 2012). ImageJ software and the Sholl analysis plug-in (Ghosh Lab) was used to determine cell cluster size at day 1 and 6. To perform the analysis, a complete image of the culture was subjected to a threshold to only include the GFP^+^ cells using the same settings for each image. To analyze the percentage of β cells that migrated towards ganglionic cells, GFP^+^ cells that overlapped with ganglionic structures (brightfield) at the beginning and end of the time lapse were analyzed.

## Supplementary Material

10.1242/develop.201009_sup1 Supplementary information
