# Supplementary material for: MafB-dependent neurotransmitter signaling promotes β cell migration in the developing pancreas
Source: Development. 2023 Mar 27;150(6):dev201009. doi: 10.1242/dev.201009 (PMC10112931; doi:10.1242/dev.201009)
Supplement: Supplementary information [file develop-150-201009-s1.pdf]

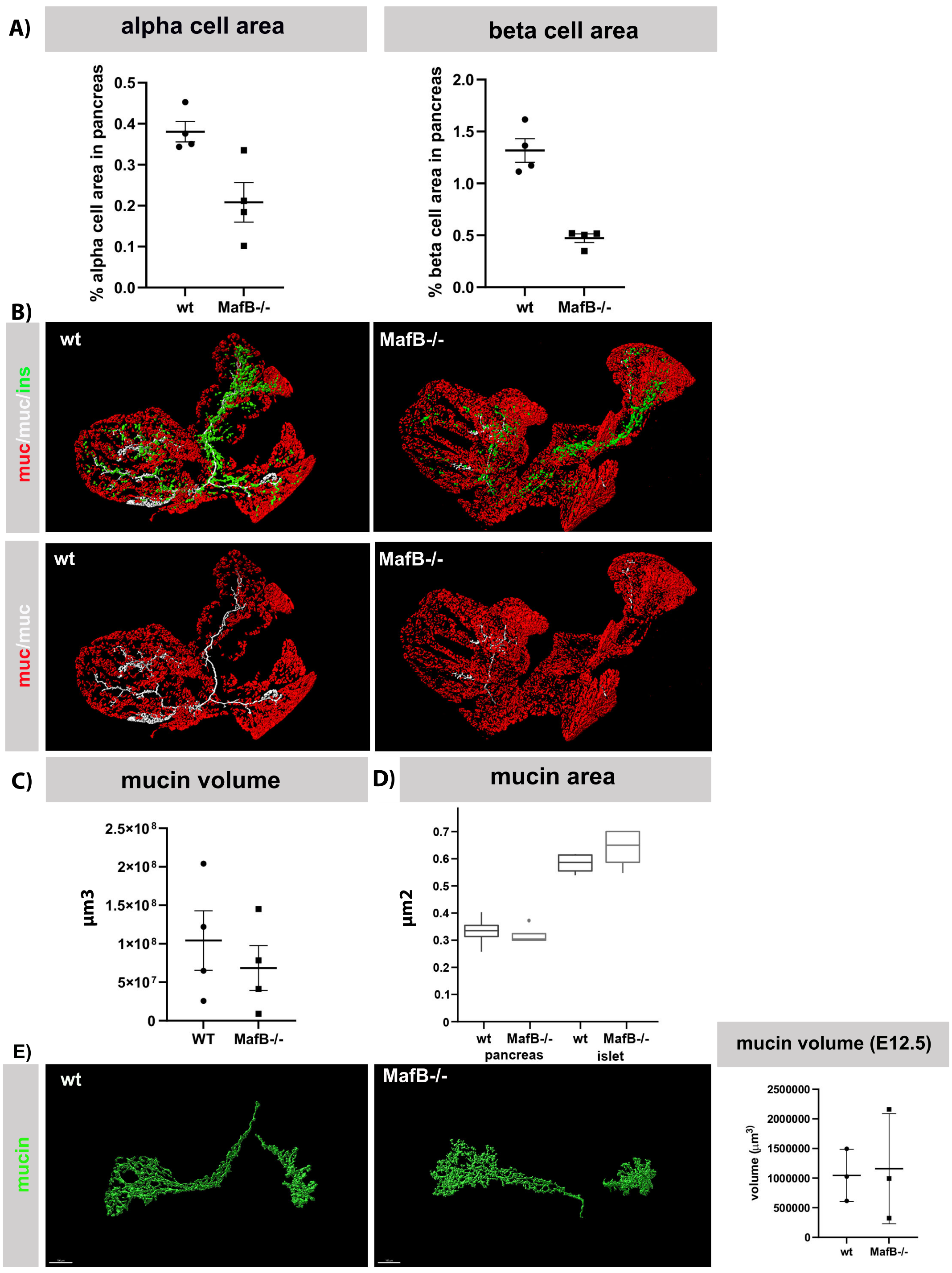

Fig. S1. Whole mount imaging of intact embryonic pancreas.

(A) Quantification of alpha and beta cell area in E18.5 wild type and *MafB*$^{-/-}$ pancreata (n=4). Alpha and beta cell area are shown as percent of total pancreatic area.

(B) Embryonic pancreas anlagen from E18.5 wild type and *MafB*$^{-/-}$ were stained with insulin and mucin and imaged by OPT and surface rendering using Imaris is shown. Large ducts (minimum length 400µm) stained with mucin were marked in white, smaller ductal structures are shown in red.

(C) Total mucin volume in wild type and *MafB*$^{-/-}$ E18.5 pancreata was assessed using Imaris imaging software (n=4).

(D) Mucin area normalized to total pancreatic and islet area of E18.5 wild type and *MafB*$^{-/-}$ pancreata (n=4).

(E) Whole mount staining of E12.5 wild type and *MafB*$^{-/-}$ pancreata with mucin (green) imaged by confocal microscopy and surface rendering and volume calculations were done using Imaris (n=3). Scale bar is 100µm.

Development • Supplementary information

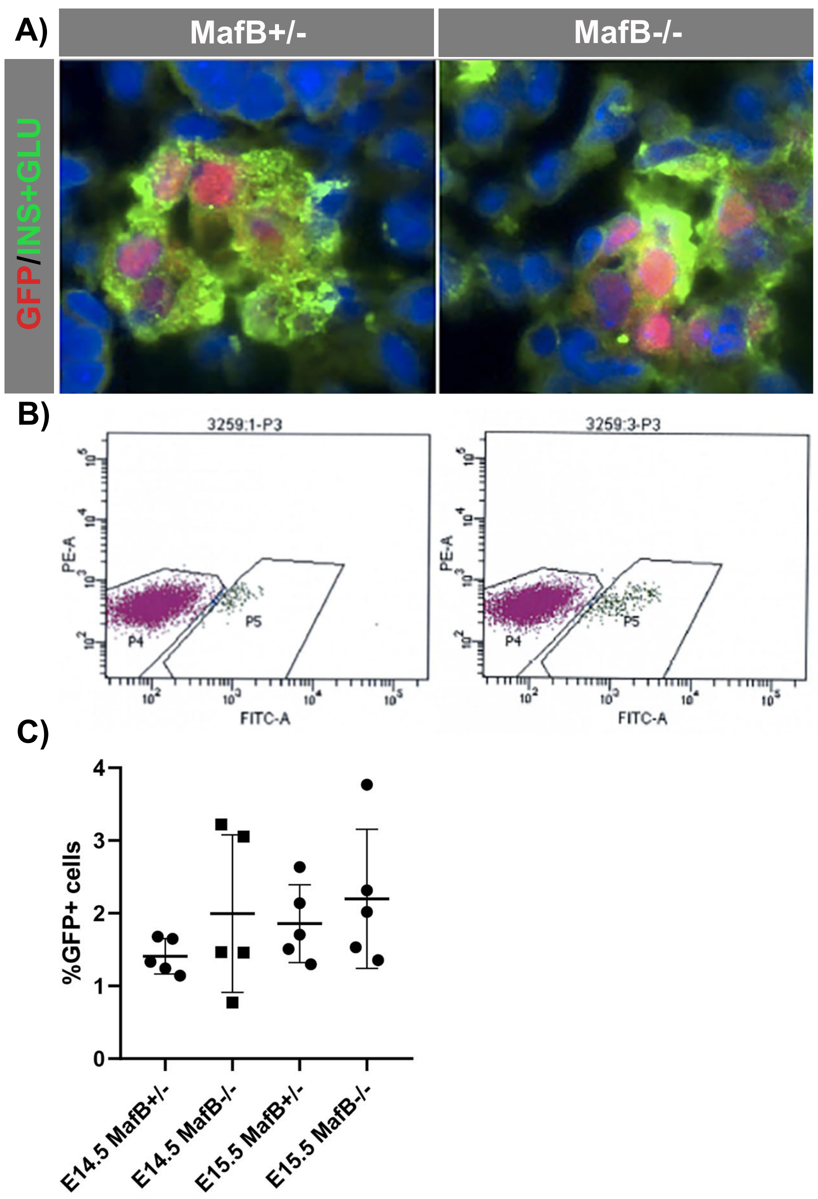

Fig. S2. Characterization of GFP expression in MafB-GFP knockin animals.

(A) Immunohistochemical staining of E14.5 *MafB*$^{+/-}$ and *MafB*$^{-/-}$ pancreas anlagen for GFP (red) and insulin+glucagon (green). Nuclei in blue. (B) FACS plot of E14.5 *MafB*$^{+/-}$ and *MafB*$^{-/-}$ pancreas showing gating strategy for GFP^{+} cell separation from negative population (x axis: FITC =GFP, y axis: PE= 7AAD viability marker). (C) Quantification of % GFP^{+} cells in E14.5 and E15.5 MafB$^{+/-}$ and MafB$^{-/-}$ pancreata (n=5).

Development • Supplementary information

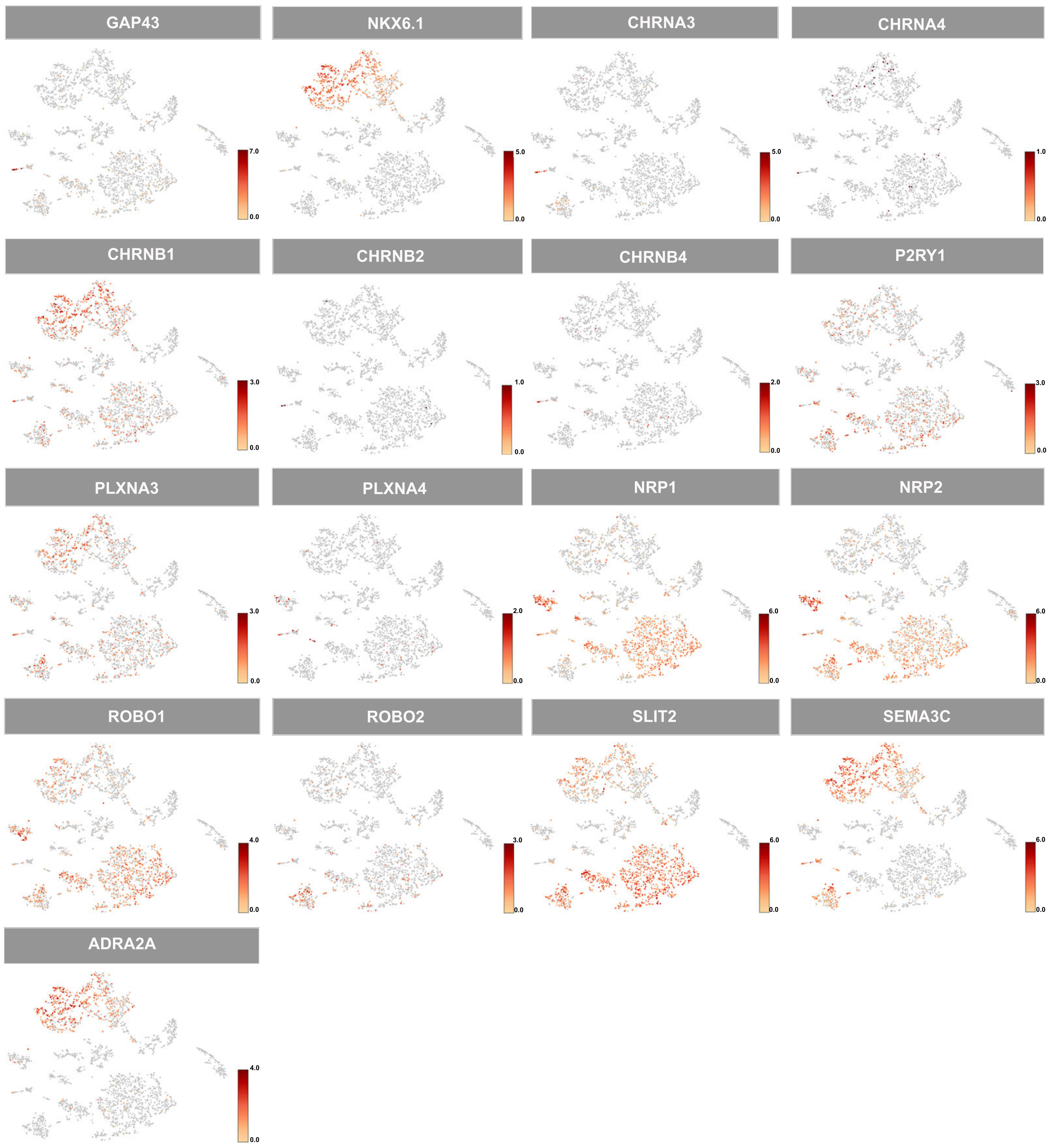

Fig. S3. Gene expression in human fetal pancreas cells

t-SNE plots showing expression of pancreatic marker (Pdx1, Nkx6.1, MafB), neural (GAP43) and neurotransmitter receptor/axon guidance genes in scRNAseq from Carnegie stage 22 human pancreas (3199 cells).

Development • Supplementary information

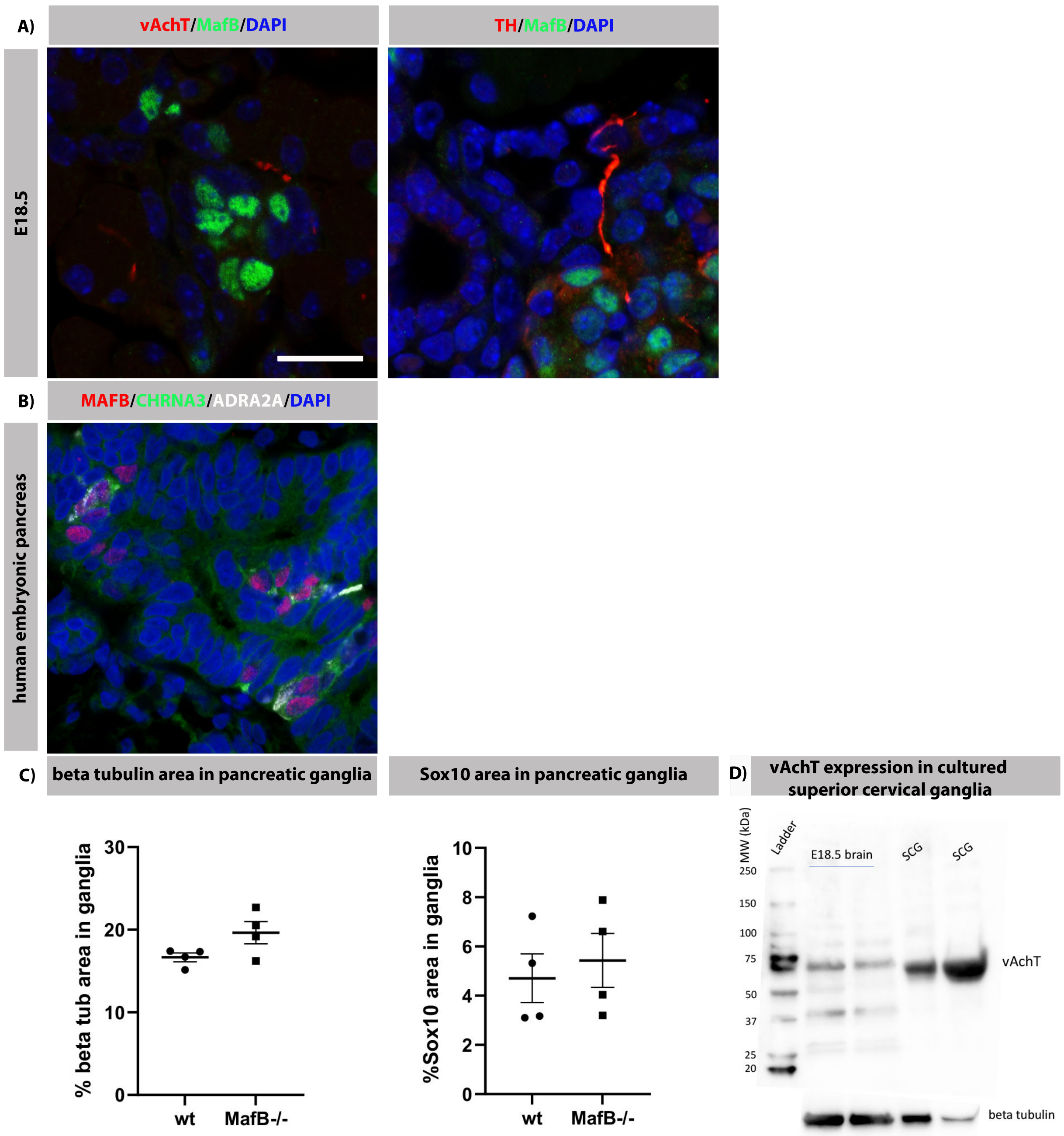

Fig. S4. **Innervation of mouse and human embryonic pancreas**

(A) Immunohistochemical stainings of E18.5 mouse pancreas with vAchT (red) or tyrosine hydroxylase (TH, red) with MafB (green) and DAPI (nuclei). Scale bar is 20µm.

(B) Immunohistochemical staining of human embryonic pancreas with MAFB (red), CHRNA3 (green), ADRA2A (with) and DAPI (nuclei).

(C) Quantification of beta III tubulin and Sox10 area in pancreatic ganglia of E18.5 wild type and *MafB*$^{-/-}$ pancreata (n=4 per genotype).

(D) Western blot showing vAchT expression in E18.5 brain and superior cervical ganglia (SCG) co-cultured for 7 days in matrigel with E18.5 pancreatic cells.

Development • Supplementary information

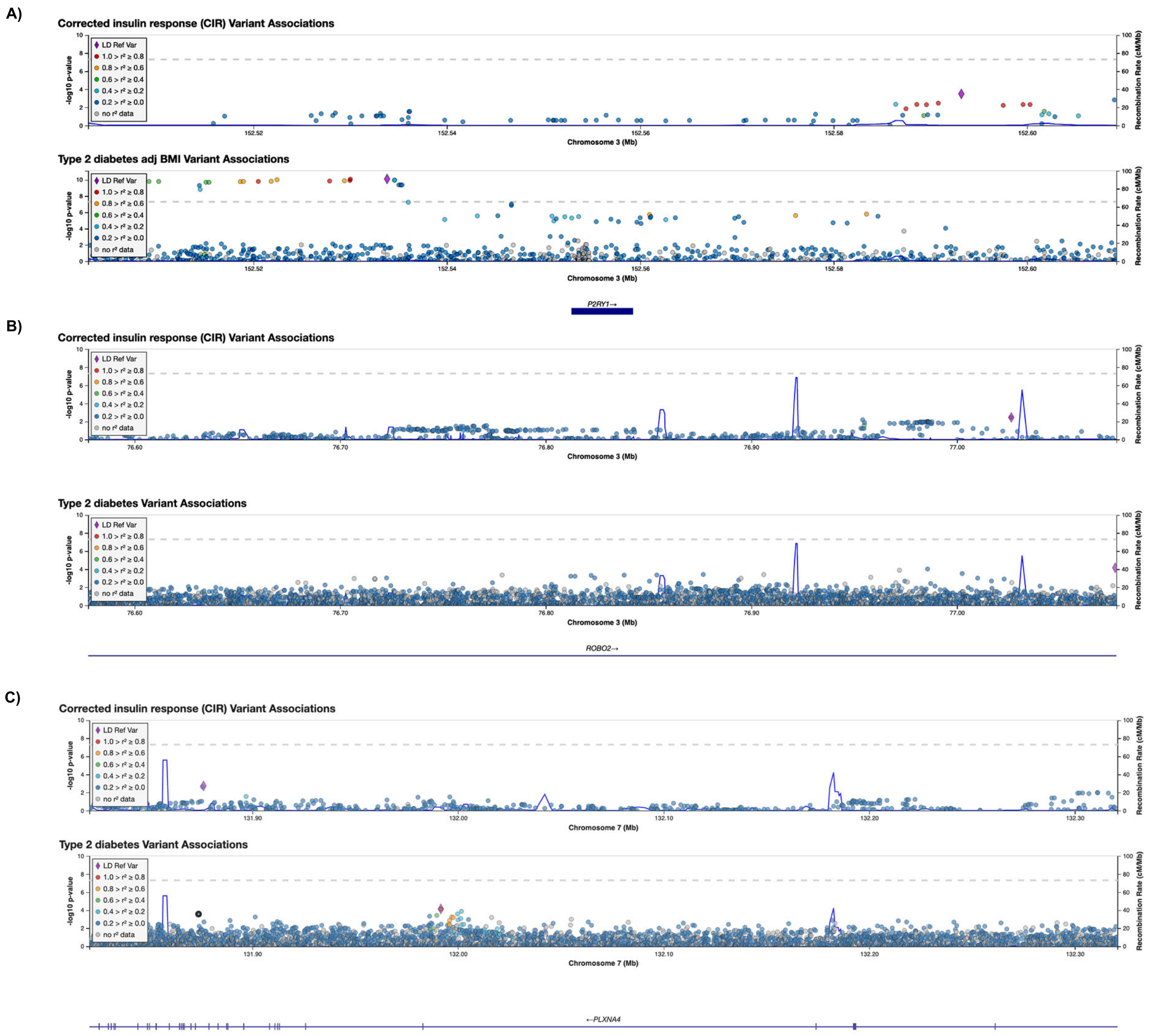

Fig. S5. Locus zoom plots for P2RY1, PLXNA4 and ROBO2

(A) Type 2 diabetes adjusted for BMI as well as suggestive signals of association for corrected insulin response (CIR) for P2RY1, (B) suggestive signals for T2D and CIR at the ROBO2 locus and (C) PLXNA4 locus. SNPs at these signals were also associated with eQTLs for their respective genes in human pancreatic islets.

Development • Supplementary information

Table S1. Gene expression analysis of MafB+/- and MafB-/- GFP-sorted E14.5 and E15.5 cells

Click here to download Table S1

Table S2. Gene ontology analysis of differentially expressed genes from MafB+/- and MafB-/- GFP+ E14.5 and E15.5 cells

Analysis Type:	PANTHER Overrepresentation Test (Released 20221013)						
Annotation Version and Release Date:	GO Ontology database DOI: 10.5281/zenodo.6799722 Released 2022-07-01						
Analyzed List:	upload_1 (Mus musculus)						
Reference List:	Mus musculus (all genes in database)						
Test Type:	FISHER						
Correction:	FDR						
GO biological process complete	Mus musculus - REFLIST (21997)	upload_1 (116)	upload_1 (expected)	upload_1 (over/under)	upload_1 (fold Enrichment)	upload_1 (raw P-value)	upload_1 (FDR)
insulin secretion (GO:0030073)	56	6	.30	+	20.32	8.85E-07	3.49E-03
peptide hormone secretion (GO:0030072)	81	6	.43	+	14.05	6.54E-06	1.03E-02
peptide secretion (GO:0002790)	86	6	.45	+	13.23	9.04E-06	1.19E-02
hormone transport (GO:0009914)	112	7	.59	+	11.85	3.14E-06	8.26E-03
protein secretion (GO:0009306)	121	7	.64	+	10.97	5.10E-06	1.15E-02
hormone secretion (GO:0046879)	104	6	.55	+	10.94	2.51E-05	2.33E-02
establishment of protein localization to extracellular region (GO:0035592)	122	7	.64	+	10.88	5.37E-06	1.06E-02
peptide transport (GO:0015833)	105	6	.55	+	10.84	2.65E-05	2.32E-02
protein localization to extracellular region (GO:0071692)	129	7	.68	+	10.29	7.61E-06	1.09E-02
negative regulation of establishment of protein localization (GO:1904950)	146	7	.77	+	9.09	1.64E-05	1.62E-02
regulation of insulin secretion (GO:0050796)	188	8	.99	+	8.07	9.26E-06	1.12E-02
regulation of blood pressure (GO:0008217)	225	8	1.19	+	6.74	3.22E-05	2.67E-02
regulation of peptide hormone secretion (GO:0090276)	232	8	1.22	+	6.54	3.97E-05	2.98E-02
regulation of peptide secretion (GO:0002791)	237	8	1.25	+	6.40	4.60E-05	2.90E-02
regulation of peptide transport (GO:0090087)	239	8	1.26	+	6.35	4.87E-05	2.95E-02
regulation of protein secretion (GO:0050708)	302	10	1.59	+	6.28	5.90E-06	1.03E-02
regulation of hormone secretion (GO:0046883)	305	9	1.61	+	5.60	4.22E-05	3.02E-02
regulation of hormone levels (GO:0010817)	584	16	3.08	+	5.20	1.00E-07	5.25E-04
trans-synaptic signaling (GO:0099537)	385	10	2.03	+	4.93	4.48E-05	2.94E-02
synaptic signaling (GO:0099536)	425	10	2.24	+	4.46	9.98E-05	4.77E-02
cell-cell signaling (GO:0007267)	894	20	4.71	+	4.24	5.66E-08	4.46E-04
regulation of establishment of protein localization (GO:0070201)	582	13	3.07	+	4.24	1.48E-05	1.67E-02
regulation of protein transport (GO:0051223)	553	12	2.92	+	4.11	4.24E-05	2.91E-02
regulation of secretion by cell (GO:1903530)	681	13	3.59	+	3.62	7.25E-05	3.57E-02
regulation of secretion (GO:0051046)	767	14	4.04	+	3.46	6.04E-05	3.53E-02
transmembrane transport (GO:0055085)	972	17	5.13	+	3.32	1.58E-05	1.66E-02
regulation of cell migration (GO:0030334)	987	16	5.20	+	3.07	6.98E-05	3.55E-02
negative regulation of multicellular organismal process (GO:0051241)	1154	18	6.09	+	2.96	3.86E-05	3.04E-02
regulation of locomotion (GO:0040012)	1092	17	5.76	+	2.95	6.64E-05	3.49E-02
regulation of biological quality (GO:0065008)	3833	54	20.21	+	2.67	5.42E-13	8.55E-09
regulation of multicellular organismal process (GO:0051239)	2989	36	15.76	+	2.28	1.81E-06	5.71E-03
biological regulation (GO:0065007)	12948	89	68.28	+	1.30	6.30E-05	3.43E-02
cellular process (GO:0009987)	15352	100	80.96	+	1.24	6.17E-05	3.48E-02

Table S3. eQTL and GWAS gene expression analysis of P2RY1, ROBO2, and PLXNA3 genes

Click here to download Table S3

Table S4. Top 20 expressed genes form individual clusters from RNA single cell sequencing form cs22 human fetal pancreas

mesenchyme		early pancreatic progenitor		erythroblasts		lymphoblasts		pancreatic progenitor (tip)		›ancreatic progenitor (trunk		vascular smooth muscle		neurons		endothelial cells	
FeatureID	FeatureName	FeatureID	FeatureName	FeatureID	FeatureName	FeatureID	FeatureName	FeatureID	FeatureName	FeatureID	FeatureName	FeatureID	FeatureName	FeatureID	FeatureName	FeatureID	FeatureName
ENSG000001(	ASPN	ENSG000001:	CENPF	ENSG000001!	HBG2	ENSG000000:	TYROBP	ENSG000001:	ERP27	ENSG000001;	KCTD16	ENSG000001(	TNNT1	ENSG000001`	GAP43	ENSG000001`	CDH5
ENSG000001(	PLAT	ENSG000001;	HIST1H1B	ENSG000002:	HBG1	ENSG000001`	C1QA	ENSG000000:	DPEP1	ENSG000001:	ERP27	ENSG000002‹	UPK3B	ENSG000001`	SCG2	ENSG000001;	CLDN5
ENSG000001!	SPARCL1	ENSG000001`	UBE2C	ENSG000001;	HBA2	ENSG000001(	AGR2	ENSG000001;	BEX5	ENSG000001(	AMBP	ENSG000002‹	SPRR2F	ENSG000001:	MAP1B	ENSG000001‹	ESAM
ENSG000001(	OGN	ENSG000000`	GTSE1	ENSG000002(	HBA1	ENSG000001!	C1QC	ENSG000001!	CPA2	ENSG000001(	CADPS	ENSG000001‹	CRB2	ENSG000001(	ELAVL4	ENSG000002‹	ECSCR
ENSG000001:	PDGFRA	ENSG000001:	KIF11	ENSG000002‹	HBB	ENSG000001!	FCER1G	ENSG000001(	SPINK1	ENSG000001(	CLDN3	ENSG000001(	PITX2	ENSG000001(	STMN2	ENSG000002(	PECAM1
ENSG000001;	PCDH18	ENSG000001‹	MKI67	ENSG000001!	ALAS2	ENSG000001(	CYBB	ENSG000000!	CPA1	ENSG000001!	SLC16A12	ENSG000001:	LGALS3	ENSG000000:	STMN4	ENSG000001`	CD34
ENSG000001(	COL6A3	ENSG000000;	BIRC5	ENSG000001(	AHSP	ENSG000002(	LST1	ENSG000001(	TM4SF4	ENSG000000:	DPEP1	ENSG000001(	PARM1	ENSG000002`	NEFL	ENSG000001`	PLVAP
ENSG000001(	SFRP1	ENSG000001:	CCNB1	ENSG000001:	HEMGN	ENSG000001(	FOLR2	ENSG000002(	MTRNR2L12	ENSG000001!	CPA2	ENSG000001`	RPRM	ENSG000001:	PRPH	ENSG000001(	EMCN
ENSG000001:	TCF21	ENSG000001:	NUSAP1	ENSG000001‹	SNCA	ENSG000001(	ITGB2	ENSG000001!	SLC16A12	ENSG000002‹	LEFTY1	ENSG000001:	DSC3	ENSG000002‹	LINC00682	ENSG000000(	TIE1
ENSG000001‹	OSR1	ENSG000001:	TOP2A	ENSG000002(	HBM	ENSG000001:	HCST	ENSG000002!	MTRNR2L8	ENSG000001(	SPINK1	ENSG000001:	ALDH1A2	ENSG000001:	TUBB2A	ENSG000001`	CLEC14A
ENSG000001:	PDLIM2	ENSG000002`	HIST1H2BH	ENSG000002:	HBE1	ENSG000001`	CD14	ENSG000000!	TGFB2	ENSG000001:	TTR	ENSG000001:	MGP	ENSG000000!	NNAT	ENSG000001`	SOX7
ENSG000001:	PDGFRB	ENSG000001:	HIST1H1A	ENSG000001`	GYPA	ENSG000000!	LYZ	ENSG000001:	NR5A2	ENSG000001(	VTN	ENSG000001;	PLA2G2A	ENSG000001(	CRABP1	ENSG000001`	A2M
ENSG000001`	NUPR1	ENSG000001!	CCNB2	ENSG000000;	HBQ1	ENSG000001(	HPGDS	ENSG000001:	RAMP1	ENSG000001:	KIF12	ENSG000001:	SULF1	ENSG000001(	TUBA1A	ENSG000001:	GIMAP4
ENSG000001(	ZEB2	ENSG000001:	CDC20	ENSG000001(	EPB42	ENSG000001;	CSF1R	ENSG000002`	CD24	ENSG000000:	CDH1	ENSG000001;	PCP4	ENSG000001`	TUBB6	ENSG000001:	KDR
ENSG000001`	CD248	ENSG000001:	CDCA3	ENSG000002!	GYPB	ENSG000001`	C1QB	ENSG000001(	AMBP	ENSG000001;	CLDN4	ENSG000001(	FRZB	ENSG000000`	CRMP1	ENSG000001(	FLT1
ENSG000000‹	ELN	ENSG000001‹	CCNA2	ENSG000001`	BPGM	ENSG000002(	AIF1	ENSG000001(	CLDN3	ENSG000001(	ONECUT1	ENSG000001:	WNT2B	ENSG000000:	CD9	ENSG000001:	MYCT1
ENSG000001:	COL5A1	ENSG000001(	KIF15	ENSG000001:	HBZ	ENSG000001(	CORO1A	ENSG000002(	TCEA3	ENSG000001:	CLDN10	ENSG000002(	CFI	ENSG000001:	DPYSL3	ENSG000000(	CALCRL
ENSG000001:	PDE1A	ENSG000000;	TPX2	ENSG000000!	BLVRB	ENSG000001:	ARHGDIB	ENSG000001`	TTR	ENSG000001:	SOX9	ENSG000001`	SERPINB9	ENSG000001:	TUBB2B	ENSG000001:	CD93
ENSG000000:	SNAI2	ENSG000001(	MAD2L1	ENSG000001!	MYL4	ENSG000001(	MNDA	ENSG000001:	KIF12	ENSG000001(	TM4SF4	ENSG000001:	BMP4	ENSG000001(	UCHL1	ENSG000001`	TCIM
ENSG000001(	COL1A2	ENSG000001:	DLGAP5	ENSG000002:	AC104389.4	ENSG000001‹	CD53	ENSG000001`	F3	ENSG000000(	ACSM3	ENSG000001`	LY6H	ENSG000001(	NEFM	ENSG000001(	ADGRL4

Development • Supplementary information
