# Supplementary material for: MafB-dependent neurotransmitter signaling promotes β cell migration in the developing pancreas
Source: Development. 2023 Mar 27;150(6):dev201009. doi: 10.1242/dev.201009 (PMC10112931; doi:10.1242/dev.201009)
Supplement: Supplementary file 1 [file develop-150-201009_review_history.pdf]

MafB-dependent neurotransmitter signaling promotes β cell migration in the developing pancreas

Sara Bsharat, Emanuela Monni, Tania Singh, Jenny K. Johansson, Kavya Achanta, Ludivine Bertonnier-Brouty, Anja Schmidt-Crhistensen, Dan Holmberg, Zaal Kokaia, Rashmi B. Prasad and Isabella Artner

DOI: 10.1242/dev.201009

Editor: James Wells

Review timeline

Original submission:	8 June 2022
Editorial decision:	6 July 2022
First revision received:	3 February 2023
Accepted:	22 February 2023

Original submission

First decision letter

MS ID#: DEVELOP/2022/201009

MS TITLE: MafB-dependent neurotransmitter signaling promotes β cell migration in the developing pancreas

AUTHORS: Emanuela Monni, Tania Singh, Sara Bsharat, Jenny Johansson, Kavya Achanta, Ludivine Bertonnier-Brouty, Anja Schmidt-Crhistensen, Dan Holmberg, Zaal Kokaia, Rashmi Prasad, and Isabella Artner

I have now received all the referees' reports on the above manuscript, and have reached a decision. The referees' comments are appended below, or you can access them online: please go to BenchPress and click on the 'Manuscripts with Decisions' queue in the Author Area.

As you will see, the referees express considerable interest in your work, but have some significant criticisms and recommend a substantial revision of your manuscript before we can consider publication. If you are able to revise the manuscript along the lines suggested, which may involve further experiments, I will be happy receive a revised version of the manuscript. Your revised paper will be re-reviewed by one or more of the original referees, and acceptance of your manuscript will depend on your addressing satisfactorily the reviewers' major concerns. Please also note that Development will normally permit only one round of major revision. If it would be helpful, you are welcome to contact us to discuss your revision in greater detail. Please send us a point-by-point response indicating your plans for addressing the referee’s comments, and we will look over this and provide further guidance.

Please attend to all of the reviewers' comments and ensure that you clearly highlight all changes made in the revised manuscript. Please avoid using 'Tracked changes' in Word files as these are lost in PDF conversion. I should be grateful if you would also provide a point-by-point response detailing how you have dealt with the points raised by the reviewers in the 'Response to Reviewers' box. If you do not agree with any of their criticisms or suggestions please explain clearly why this is so.

© 2023. Published by The Company of Biologists under the terms of the Creative Commons Attribution License (https://creativecommons.org/licenses/by/4.0/).

Reviewer 1

Advance summary and potential significance to field

The transcription factor *Mafb* plays important but different roles in both murine and human pancreatic endocrine β-cell development. In the mouse, *Mafb* is required for terminal differentiation of β-cell but then *Mafa* is expressed and become dominant. In contrast, MAFB in humans continues to be expressed in adult β-cells and is essential for producing glucose-responsive β-cells from hPSCs. In the current study, the authors show that *Mafb* regulates the expression of genes encoding receptors for neurotransmitters and axon guidance in mice. These findings suggest that *Mafb* could regulate pancreatic endocrine cell development through effects on cellular migration.

Comments for the author

In this study, the authors use global *Mafb* KO mice and microarray analysis of FAC-sorted *Mafb*-positive cells to assess defects in nascent β-cell migration. These analyses suggest that *Mafb* is important for the expression of several neurotransmitter and axon guidance receptors, including nicotinic acetylcholine receptors. Using a co-culture of developing β-cells with nerve ganglia, the authors then show that nicotinic receptor signaling promotes β-cell migration towards nerves. However, while single cell (sc)-qPCR suggests that the receptor genes are predominantly expressed in β-cells, immunostaining shows that many of the genes are also expressed in alpha-cells. scRNA-seq analysis of human fetal pancreata indicates that several neurotransmitter and axon guidance receptors are expressed in human developing endocrine cells, and a re-analysis of previously published datasets of human hPSCs, islets and eQTLs suggests that the expression of neurotransmitters and axon guidance receptor genes is regulated by MAFB in humans, and that the regulation is relevant to adult islet function.

While the findings in this study are of potential interest, the results as currently presented fall short of compelling due in substantial part to how they are presented and to some variabilities in the data. The authors need to better connect the very divergent parts of the manuscript, provide a more explicit rationale for performing various analyses, and improve the quality of some of the data.

Additional comments:

1. The paper would benefit from additional proofreading. For example, in several instances, it is written “neurotransmitter receptor gene expression” in the reference to multiple genes.

2. The role of *Mafb* in the specification and late stages of β- and β-cells differentiation (E15.5 - E18.5 in mice) is referred to as “maturation”.
It would be better to refer to this process as terminal differentiation to avoid confusion and to distinguish this stage from the functional maturation of β-cells which takes place only after birth.

3. The study uses global *Mafb* KO mice to quantify the differences in developing β-cell migration and ductal morphogenesis. This complicates interpretation of the results since *Mafb* is not only expressed in pancreatic endocrine cells, it is also expressed in mesenchymal cells, neural cells, and vascular and blood cells during development and also contributes to the development of these cell lineages. Indeed, Fig. 4F clearly demonstrates that MAFB is expressed in these tissues in the fetal human pancreas. Although the authors mention that the pancreata are “indistinguishable” between I>Mafb+/- and *Mafb-/-* mice, there may be differences in the function of and signaling from mesenchymal, vascular, and neural cells in *Mafb* KO that might affect ductal cell development and endocrine cell migration.

4. It is not clear what kind of ductal structures were used for quantification presented in Fig.1B. Only large ducts are shown in Fig 1A. Were the small ducts shown in red in Fig.S1 used for quantification, large ducts or all ducts used for quantification? This needs to be specified in the Method description. The mucin-positive volume quantifications at the bottom of Fig.S1 do not appear to be significant. This should be indicated on the graph or in the figure legend. Moreover,

© 2023. Published by The Company of Biologists under the terms of the Creative Commons Attribution License (https://creativecommons.org/licenses/by/4.0/).

the text in the Results stating that “smaller lumen was present and … total ductal volume was impaired” should be removed or it should clearly state that the observed differences were not significant.

5. When global expression analysis is described, it should be indicated that they were obtained using microarrays. Microarray data for expression analysis between *Mafb*+/- and *Mafb*-/- GFP-positive cells are not presented in the paper. The supplemental table containing lists of all differentially expressed genes including expression values, fold change, and statistics should be included in the study. How many genes were changed significantly? The reader is only informed that selected genes have changed. What about all the other genes that are expressed in β-cells? Why were only specific neurotransmitter and axon guidance receptors genes chosen to present? Did other neurotransmitter receptors also change? Are other groups of genes up-regulated and down-regulated? Was gene ontology enrichment analysis performed on differentially expressed genes?

6. The paragraph describing the Fluidigm sc-qPCR should state at what age the GFP+ cells were analyzed. It is not clear from Fig.2B how the authors made a conclusion that the “expression of neurotransmitter and axon guidance receptors genes was predominantly observed in *Mafb*+/- β-cells, but reduced in *Mafb*-/- cells”? For example, Fig. 2A shows that the expression of Chrnb2 and Plxna3 are increased in *Mafb* KO cells at E15.5. A callout for Fig.2A was not found in the text.

7. Changes in the immunofluorescence staining presented in Fig 3. are not always discernable. No insets are seen in panels E and F. Panels O and P show that Plxna3 is decreased in the *Mafb* KO cells but the staining does appear to be increased in Fig. 2A.

8. The inclusion in the study of human scRNA-seq data is interesting however, it does not add much to the main message of the study which is centered on the regulation of neurotransmitter and axon guidance receptor genes.

9. In Fig 4 the gray shading at the top is too dark and makes it hard to see green text in panels with immunofluorescence. Overall, the images are not easily interpretable, the DAPI staining seems to be oversaturated and makes it hard to see staining in other channels. The insets in Fig. 4L and M do not show Pdx1, the inset on Fig. 4N is missing. The text near the pie charts in Fig.4K is very hard to read and the font should be increased.

10. It is not clear why the authors chose to use MIP-GFP-positive-cells in the nerve and β-cell co-culture experiments and at what age the MIP-GFP-positive β-cells were isolated for the co-culture experiments? The age should be mentioned in the corresponding Results and Methods section. Since the main message that this study strives to convey is that *Mafb* regulates neuronal-directed signaling events that promote β-cell migration during development, the co-culture experiment should also use GFP-positive FACS purified *Mafb*-expressing cells from *Mafb*+/- and *Mafb*-/- animals (similar to those used for microarray and ss-qPCR) that were used for microarrays and sc-qPCR.

<u>Reviewer 2</u>

Advance summary and potential significance to field

In this study, Monni et al report a novel role for the MafB transcription factor in islet embryonic development, which involves regulating the neurotransmitter sensing of cells. More specifically, the authors describe how endocrine beta cell migration and clustering depend on nicotinic signaling, a process that might be mediated by islet-nerve cell communication.

Overall, the manuscript is well-written and the results are interesting and of high quality. The results would be of interest to researchers in the field of beta cell developmental biology.

Comments for the author

Some methodological issues need to be clarified, and additional experimentation would be necessary to strengthen the main conclusions of the study as well as to increase the impact of the paper.

© 2023. Published by The Company of Biologists under the terms of the Creative Commons Attribution License (https://creativecommons.org/licenses/by/4.0/).

Specific comments:
1. The finding that loss of MafB disrupts ductal morphogenesis is very interesting, although somewhat unexpected considering that MafB is not expressed in embryonic ductal cells.
The authors argue that ductal morphogenesis defects might be a secondary defect due to impaired endocrine cell migration. Perhaps one of the best studies that supports this theory is the impaired ductal morphogenesis of Ngn3 mutant embryos (Magenheim et al. Developmental Biology Volume 359, Issue 1, 1 November 2011 Pages 26-36) and probably should be discussed in the manuscript.

However, the characterization of the ductal defects of MafB embryonic pancreata (Figure 1) is not very thorough. First, the number of embryos analyzed is low and a high variability in the data is observed (Fig.S1). Also, it is important to describe whether the analyzed embryos come from independent litters. Second, in Figure 1B is not clear how beta cell distance from ducts was measured. I assume that it was measured only from large ducts (> 400 um)? It should be properly described in the text. Similarly, it needs be defined what “mucin structures” are in Fig. S1 (large ducts?). Third, the authors claimed that islet cells in MafB pancreata “appeared to be organized in smaller clusters” but no quantification is provided. Finally, a deeper analysis of ductal morphogenesis should be performed including dilation of the ducts and branching of the ductal tree, as described by Magenheim et al. An analysis of the ductal tree at earlier embryonic time points (e.g. at E12.5 before the secondary transition) could also help to support the author’s conclusion that ductal morphogenesis defects are due to impaired endocrine cell migration.

2. Loss of MafB impairs neurotransmitter and axon guidance receptor gene expression in beta cells, yet surprisingly, total islet innervation is unaffected. More details about the quantification of islet innervation should be provided. Precise quantification of islet innervation can be finicky, and it is necessary to examine a good sampling of the pancreas. In this regard, how many sections per pancreas were analyzed? How was “islet” defined when measuring “islet innervation in E18.5 embryos? Isn’t islet size diminished in MafB pancreata? Maybe measuring total pancreatic innervation in this particular case might be more informative?
In addition to nerve fibers, neural cell bodies (intrapancreatic ganglia) are found in the pancreas and might be potentially affected by loss of MafB. The authors should examine whether the number of intrapancreatic glial and neuronal cells is affected in MafB mutants. The authors argue that the continued expression of NGF in MafB mutant pancreata could explain the lack of defects in islet innervation. Another growth factor critical for islet innervation is the glial-derived neurotrophic factor (GDNF) is (Development 2013 Sep;140(17):3669-79). Is pancreatic GDNF expression affected in MafB mutants?

3. Regarding the co culture experiments with cervical ganglia shown in Figure 5 why haven’t the authors also analyzed beta cell migration in MafB mutants? These ex vivo studies are a useful assay to directly test the role of MafB in beta cell migration.

Reviewer 3

Advance summary and potential significance to field

In this study, Monni and colleagues propose that a population of endocrine progenitor cells (defined by expression of MafB) express neurotransmitter and axon guidance receptor genes that serve to instruct islet morphogenesis. The authors use a combination of mouse genetics, single-cell sequencing, and imaging approaches to elucidate a function for the MafB transcription factor in regulating islet-nerve cell communication, and report cholinergic signaling as a key mechanism for beta-cell migration and clustering. Defining how islet endocrine cells respond to spatial extrinsic cues, especially those coming from peripheral nerves is an important, and understudied, topic in islet biology. In this context, the study addresses an area of high significance

Comments for the author

In this study, Monni and colleagues propose that a population of endocrine progenitor cells (defined by expression of MafB) express neurotransmitter and axon guidance receptor genes that serve to instruct islet morphogenesis. The authors use a combination of mouse genetics, single-cell sequencing, and imaging approaches to elucidate a function for the MafB transcription factor in

© 2023. Published by The Company of Biologists under the terms of the Creative Commons Attribution License (https://creativecommons.org/licenses/by/4.0/).

regulating islet-nerve cell communication, and report cholinergic signaling as a key mechanism for beta-cell migration and clustering. Defining how islet endocrine cells respond to spatial extrinsic cues, especially those coming from peripheral nerves is an important, and understudied, topic in islet biology. The study reports on an interesting topic and the data, specifically, the sequencing studies, are well-done. However, a major concern with the study is the conclusion that the defects of islet morphology in $MafB^{-/-}$ mice are due to the reduced expression of nicotinic receptors. The study is largely correlative, relying predominantly on sequencing analyses and some imaging to draw conclusions about nerve function and neuronal signaling events in beta-cell migration and clustering. The manuscript would benefit from additional analyses to show directly the spatial relationship between autonomic nerves (sympathetic/parasympathetic) and the MafB-positive endocrine population, as well as to better support the conclusion that beta-cells migrate/cluster in response to cholinergic signaling.

Major concerns:

1. The spatial relationship between sympathetic/parasympathetic nerves and MafB progenitor population should be better investigated by performing immunostaining for TH (sympathetic) or ChAT/VAChT in mouse and human pancreatic tissues. The images in Fig 4I, J are not very clear and it is hard to distinguish signal in the nerves from cell bodies in the tissue sections.

2. The authors propose a role for nicotinic signaling emanating from Acetylcholine (Ach) released by parasympathetic fibers in regulating beta-cell clustering. This conclusion should be supported by additional analyses, specifically genetic tools to manipulate cholinergic signaling, such as AchR deletion/kd in MafB+ cells or ablation of parasympathetic nerves in vivo. It is particularly puzzling that the authors used the superior cervical ganglia (SCG) as a source of Ach in the nerve-islet co-cultures. SCG nerves are adrenergic and would be expected to secrete Norepinephrine in the co-cultures. The primary source of Ach in the SCG will be the pre-ganglionic nerves which would be lost in ganglia dissection. The authors should be using parasympathetic ganglia in the co-culture system.

3. is interesting that adrenergic and cholinergic receptors are expressed in the same MafB+ population based on sequencing, suggesting that they respond to both cholinergic and adrenergic signals. To my knowledge, this is rare. The sequencing studies should be supported by co-localization using immunohistochemistry to show that this endocrine population expresses both cholinergic and adrenergic signaling machinery.

4. A revised model of islet morphogenesis suggests that instead of delamination of individual endocrine cells from the ducts, migration, and aggregation, islets develop as long interconnected chains along the ducts, which then undergo fission. The authors should discuss their findings of nerve-islet communication in the context of the revised understanding of islet formation (see recent review by Adams and Blum 2022).

Minor concerns:

1. The Methods section is missing the details for optical projection tomography or immunohistochemical analyses

2. Overall, there should be more details of antibody validation, specifically against the acetylcholine and adrenergic receptors, either from the literature or by the authors themselves.

3. For Fig. 4H, the large green spots look too large to be nuclear labeling. Could the authors distinguish the MafB-immunopositive signal from the non-specific staining?

© 2023. Published by The Company of Biologists under the terms of the Creative Commons Attribution License (https://creativecommons.org/licenses/by/4.0/).

First revision

Author response to reviewers' comments

A description of the changes made in response to the reviewers' comments are detailed below, with modifications to the manuscript body highlighted in red:

Reviewer 1

Major Points:

Comment: While the findings in this study are of potential interest, the results as currently presented fall short of compelling due in substantial part to how they are presented and to some variabilities in the data. The authors need to better connect the very divergent parts of the manuscript, provide a more explicit rationale for performing various analyses, and improve the quality of some of the data.

Response: *We have edited the manuscript to better connect the different parts and included several new experiments requested by the reviewers.*

Additional comments:

Comment 1: The paper would benefit from additional proofreading. For example, in several instances, it is written "neurotransmitter receptor gene expression" in the reference to multiple genes.

Response: *We have updated the manuscript accordingly.*

Comment 2: The role of *Mafb* in the specification and late stages of β- and β-cells differentiation (E15.5 -E18.5 in mice) is referred to as "maturation". It would be better to refer to this process as terminal differentiation to avoid confusion and to distinguish this stage from the functional maturation of β- cells which takes place only after birth.

Response: *The manuscript has been changed according to the reviewer's suggestion.*

Comment 3: The study uses global *Mafb* KO mice to quantify the differences in developing β-cell migration and ductal morphogenesis. This complicates interpretation of the results since *Mafb* is not only expressed in pancreatic endocrine cells, it is also expressed in mesenchymal cells, neural cells, and vascular and blood cells during development and also contributes to the development of these cell lineages. Indeed, Fig. 4F clearly demonstrates that MAFB is expressed in these tissues in the fetal human pancreas. Although the authors mention that the pancreata are "indistinguishable" between I>Mafb+/- and *Mafb-/-* mice, there may be differences in the function of and signaling from mesenchymal, vascular, and neural cells in *Mafb* KO that might affect ductal cell development and endocrine cell migration.

Response: *Previous studies have shown that MafB expression is critical for development of multiple cell types including macrophages and blood vessel. However, protein expression in the pancreas was predominantly detected in endocrine cells (Artner et al., 2006; Nishimura et al., 2006). When sorting cells from MafB$^{+/-}$ pancreata (GFP knockin to the MafB locus) we identified single macrophages, but all other sorted GFP+ cells expressed pancreatic endocrine cell markers (Fig.2B). Moreover, when assessing MafB expression in single cell RNA sequencing data from wild type E15 pancreas (see below), the majority of the MafB+ cells were endocrine progenitor and immune cells suggesting that MafB's main function is in endocrine progenitor cells. This is further supported by a study using conditional MafB knock out mice (MafB deletion in the pancreatic epithelium using Pdx1-driven Cre) which also described a ~50% reduction of insulin and glucagon producing cells at E15.5 (Conrad et al., 2016) arguing for MafB's function being cell-autonomous in developing endocrine progenitor cells. Moreover, we have assessed ductal morphology at E12.5 (prior to onset of endocrine cell differentiation) and did not observe differences between wild*

© 2023. Published by The Company of Biologists under the terms of the Creative Commons Attribution License (https://creativecommons.org/licenses/by/4.0/).

type and $MafB^{-/-}$ samples (Fig. S1) suggesting that the observed phenotype is only emerging after the onset of endocrine cell differentiation.

NOTE: We have removed unpublished data that had been provided for the referees in confidence.

Comment 4: It is not clear what kind of ductal structures were used for quantification presented in Fig.1B. Only large ducts are shown in Fig 1A. Were the small ducts shown in red in Fig.S1 used for quantification, large ducts or all ducts used for quantification? This needs to be specified in the Method description. The mucin-positive volume quantifications at the bottom of Fig.S1 do not appear to be significant. This should be indicated on the graph or in the figure legend. Moreover, the text in the Results stating that "smaller lumen was present and … total ductal volume was impaired" should be removed or it should clearly state that the observed differences were not significant.

Response: *All ductal structures were used for assessing the distance between ducts and beta cells. We have added a sentence in the figure legend clarifying that ductal volume is not impaired in MafB-/- pancreata (Fig.S1) and modified results and methods sections to better describe the analysis and phenotype. We have also added an additional histology analysis showing that ductal histology in the islet is impaired with less branching points when compared to the wild type (Fig.1 and Fig.S1)*

Comment 5: When global expression analysis is described, it should be indicated that they were obtained using microarrays. Microarray data for expression analysis between *Mafb*+/- and *Mafb*-/- GFP-positive cells are not presented in the paper. The supplemental table containing lists of all differentially expressed genes including expression values, fold change, and statistics should be included in the study. How many genes were changed significantly? The reader is only informed that selected genes have changed. What about all the other genes that are expressed in β-cells? Why were only specific neurotransmitter and axon guidance receptors genes chosen to present? Did other neurotransmitter receptors also change? Are other groups of genes up-regulated and down-regulated? Was gene ontology enrichment analysis performed on differentially expressed genes?

Response: *We have included a complete list of genes that were changes at the different time points as supplementary data (Supplementary Table 1) and performed a gene ontology analysis (Supplementary Table 2). As indicated in the text, known MafB target genes important for beta cell function were also changed in $MafB^{-/-}$ samples and differentially expressed genes were associate with biological processes hormone secretion, cell migration and synaptic signaling.*

Comment 6: The paragraph describing the Fluidigm sc-qPCR should state at what age the GFP+ cells were analyzed. It is not clear from Fig.2B how the authors made a conclusion that the "expression of neurotransmitter and axon guidance receptors genes was predominantly observed in *Mafb*+/- β-cells, but reduced in *Mafb*-/- cells"? For example, Fig. 2A shows that the expression of Chrnb2 and Plxna3 are increased in *Mafb* KO cells at E15.5. A callout for Fig.2A was not found in the text.

Response: *We have updated to include information on the stage of the sorted cells and specified which genes were downregulated.*

Comment 7: Changes in the immunofluorescence staining presented in Fig 3. are not always discernable. No insets are seen in panels E and F. Panels O and P show that Plxna3 is decreased in the *Mafb* KO cells but the staining does appear to be increased in Fig. 2A.

Response: *We have included inserts for all stainings and added arrows to highlight the increase of $PlxnA3^{+}$ cells in the knockout.*

Comment 8: The inclusion in the study of human scRNA-seq data is interesting, however, it does not add much to the main message of the study which is centered on the regulation of neurotransmitter and axon guidance receptor genes.

© 2023. Published by The Company of Biologists under the terms of the Creative Commons Attribution License (https://creativecommons.org/licenses/by/4.0/).

***Response:** Human scRNA sequencing data, as well as immunohistochemistry were included to show that neurotransmitter receptor genes were also expressed in human embryonic pancreas and to assess if a similar relationship between MafB and neurotransmitter receptor genes expression can be observed in both mouse and human developing endocrine progenitor cells.*

Comment 9: In Fig 4 the gray shading at the top is too dark and makes it hard to see green text in panels with immunofluorescence. Overall, the images are not easily interpretable, the DAPI staining seems to be oversaturated and makes it hard to see staining in other channels. The insets in Fig. 4L and M do not show Pdx1, the inset on Fig. 4N is missing. The text near the pie charts in Fig.4K is very hard to read and the font should be increased.

***Response:** We have modified the figure according to the suggestions and removed the DAPI channel to better visualize the hormone and neurotransmitter receptor stainings, as well as included inserts and updated fig. Fig.4K.*

Comment 10: It is not clear why the authors chose to use MIP-GFP-positive-cells in the nerve and β-cell co-culture experiments and at what age the MIP-GFP-positive β-cells were isolated for the co-culture experiments? The age should be mentioned in the corresponding Results and Methods section. Since the main message that this study strives to convey is that *Mafb* regulates neuronal-directed signaling events that promote β-cell migration during development, the co-culture experiment should also use GFP-positive FACS purified*Mafb*-expressing cells from *Mafb*+/- and *Mafb*-/- animals (similar to those used for microarray and ss-qPCR) that were used for microarrays and sc-qPCR.

Response: We agree that using MafB mutant cells would have been optimal. We have tried to use MafB$^{+/-}$ and $^{-/-}$ cells for this analysis, but unfortunately the GFP expression from the MafB locus was not bright enough to visualize in the confocal microscope to perform live cell imaging. Thus we have decided to use MIP-GFP transgenic cells to test the effect of blocking nicotinic signal on wild type beta cells. MIP-GFP producing cells were isolated from E18.5 pancreata.

Reviewer 2 Advance Summary and Potential Significance to Field:
In this study, Monni et al report a novel role for the MafB transcription factor in islet embryonic development, which involves regulating the neurotransmitter sensing of cells. More specifically, the authors describe how endocrine beta cell migration and clustering depend on nicotinic signaling, a process that might be mediated by islet-nerve cell communication.

Overall, the manuscript is well-written and the results are interesting and of high quality. The results would be of interest to researchers in the field of beta cell developmental biology.

Specific comments:

Comment 1: The finding that loss of MafB disrupts ductal morphogenesis is very interesting, although somewhat unexpected considering that MafB is not expressed in embryonic ductal cells. The authors argue that ductal morphogenesis defects might be a secondary defect due to impaired endocrine cell migration. Perhaps one of the best studies that supports this theory is the impaired ductal morphogenesis of Ngn3 mutant embryos (Magenheim et al. Developmental Biology Volume 359, Issue 1, 1 November 2011, Pages 26-36) and probably should be discussed in the manuscript. However, the characterization of the ductal defects of MafB embryonic pancreata (Figure 1) is not very thorough. First, the number of embryos analyzed is low and a high variability in the data is observed (Fig.S1). Also, it is important to describe whether the analyzed embryos come from independent litters. Second, in Figure 1B is not clear how beta cell distance from ducts was measured. I assume that it was measured only from large ducts (> 400 um)? It should be properly described in the text. Similarly, it needs be defined what “mucin structures” are in Fig. S1 (large ducts?). Third, the authors claimed that islet cells in MafB pancreata “appeared to be organized in smaller clusters” but no quantification is provided. Finally, a deeper analysis of ductal morphogenesis should be performed, including dilation of the ducts and branching of the ductal tree, as described by Magenheim et al. An analysis of the ductal tree at earlier embryonic time points (e.g. at E12.5 before the secondary transition) could also help to support the author’s

© 2023. Published by The Company of Biologists under the terms of the Creative Commons Attribution License (https://creativecommons.org/licenses/by/4.0/).

conclusion that ductal morphogenesis defects are due to impaired endocrine cell migration.

Response: *We have updated the manuscript to include a discussion of Neurog3's role in ductal morphogenesis. We have also extended the analysis of ductal and islet morphology to include measurements of branching points, mucin area, and size distribution of ducts, as well as alpha and beta cell area in wild type and MafB mutant pancreata (Fig.1 and Fig.S1). This analysis has been performed on sections which enabled us to have an automated recognition of islet area. The analysis shows that ductal structures within the islet area have less branching points and are smaller in MafB mutant pancreata, while ductal structures in the exocrine portion of the pancreas were indistinguishable between wild type and knock out embryos. We have also performed a whole mount analysis of E12.5 pancreata which showed that the ductal network at this earlier stage is not altered in the knock out arguing that the phenotype only becomes evident after the onset of the endocrine cell differentiation. We have also included information that distance from duct to islet cells was performed from all ductal structures (Fig.1B), how many different litters were included in the 3D analysis and the color coding of ductal structures in Fig. S1.*

Comment 2: Loss of MafB impairs neurotransmitter and axon guidance receptor gene expression in beta cells, yet surprisingly, total islet innervation is unaffected. More details about the quantification of islet innervation should be provided. Precise quantification of islet innervation can be finicky, and it is necessary to examine a good sampling of the pancreas. In this regard, how many sections per pancreas were analyzed? How was "islet" defined when measuring "islet innervation in E18.5 embryos? Isn't islet size diminished in MafB pancreata? Maybe measuring total pancreatic innervation in this particular case might be more informative? In addition to nerve fibers, neural cell bodies (intrapancreatic ganglia) are found in the pancreas and might be potentially affected by loss of MafB. The authors should examine whether the number of intrapancreatic glial and neuronal cells is affected in MafB mutants. The authors argue that the continued expression of NGF in MafB mutant pancreata could explain the lack of defects in islet innervation. Another growth factor critical for islet innervation is the glial- derived neurotrophic factor (GDNF) is (Development 2013 Sep;140(17):3669-79). Is pancreatic GDNF expression affected in MafB mutants?

Response: *We agree and have performed quantification of total pancreatic innervation, intra-pancreatic ganglia and glial cell areas, which were all unchanged in $MafB^{-/-}$ pancreata when compared to wild type tissue. These data are now included in the manuscript (Fig. 4 and Fig S4).We have also checked if GDNF expression was significantly changed in $MafB^{-/-}$ cells and do not see a change (logFC = 0.1825, P =0.21).*

Comment 3: Regarding the co-culture experiments with cervical ganglia shown in Figure 5, why haven't the authors also analyzed beta cell migration in MafB mutants? These ex vivo studies are a useful assay to directly test the role of MafB in beta cell migration.

Response: *We agree that this experimental set up would have been very useful. Unfortunately MafB-driven GFP expression in $MafB^{+/-}$ and $MafB^{-/-}$ was not bright enough to visualize using a fluorescence microscope. Thus we chose to use MIP-GFP mice to assess if blocking of nicotinic receptor function was impairing cell migration in wild type beta cells.*

Reviewer 3 Comments for the Author:

In this study, Monni and colleagues propose that a population of endocrine progenitor cells (defined by expression of MafB) express neurotransmitter and axon guidance receptor genes that serve to instruct islet morphogenesis. The authors use a combination of mouse genetics, single-cell sequencing, and imaging approaches to elucidate a function for the MafB transcription factor in regulating islet-nerve cell communication, and report cholinergic signaling as a key mechanism for beta-cell migration and clustering. Defining how islet endocrine cells respond to spatial extrinsic cues, especially those coming from peripheral nerves is an important, and understudied, topic in islet biology. The study reports on an interesting topic and the data, specifically, the sequencing studies, are well-done. However, a major concern with the study is the conclusion that the defects of islet morphology in MafB-/- mice are due to the reduced expression of nicotinic receptors. The study is largely correlative, relying predominantly on sequencing analyses and some imaging to draw conclusions about nerve function and neuronal signaling events in beta-cell

© 2023. Published by The Company of Biologists under the terms of the Creative Commons Attribution License (https://creativecommons.org/licenses/by/4.0/).

migration and clustering. The manuscript would benefit from additional analyses to show directly the spatial relationship between autonomic nerves (sympathetic/parasympathetic) and the MafB-positive endocrine population, as well as to better support the conclusion that beta-cells migrate/cluster in response to cholinergic signaling.

Major concerns:

Comment 1: The spatial relationship between sympathetic/parasympathetic nerves and MafB progenitor population should be better investigated by performing immunostaining for TH (sympathetic) or ChAT/VAChT in mouse and human pancreatic tissues. The images in Fig 4I, J are not very clear and it is hard to distinguish signal in the nerves from cell bodies in the tissue sections.

Response: *We have performed immunohistochemical stainings for TH, vAchT, and MafB in human and mouse embryonic pancreas (Fig.4 and Fig.S4).*

Comment 2: The authors propose a role for nicotinic signaling emanating from Acetylcholine (Ach) released by parasympathetic fibers in regulating beta-cell clustering. This conclusion should be supported by additional analyses, specifically genetic tools to manipulate cholinergic signaling, such as AchR deletion/kd in MafB+ cells or ablation of parasympathetic nerves in vivo. It is particularly puzzling that the authors used the superior cervical ganglia (SCG) as a source of Ach in the nerve-islet co-cultures. SCG nerves are adrenergic and would be expected to secrete Norepinephrine in the co-cultures. The primary source of Ach in the SCG will be the pre-ganglionic nerves which would be lost in ganglia dissection. The authors should be using parasympathetic ganglia in the co-culture system.

Response: *We agree that it would be very interesting to genetically manipulate AchR receptor expression in MafB$^+$ cells and assess beta cell migration. Unfortunately, several different nicotinic receptor subunits are simultaneously expressed in mouse and human MafB$^+$ cells (ChrnA3, ChrnA4, ChrnB2, ChrnB4 and ChrnB1), making it difficult to delete nAchR function genetically. Thus blocking all nAchR activity with mecamylamine was chosen to assess nAchR function.*

We agree that parasympathetic ganglia would be a superior source of acetylcholine, however these developing intrapancreatic ganglia are too small to dissect for co-culture experiments. Previous studies by (Mochida et al., 1994; Patterson and Chun, 1977) have shown that sympathetic ganglia start to express enzymes/transporters for Ach production and secretion when cultured. We have now included a Western blot showing that vAchT protein is readily detectable in superior cervical ganglia co-cultured with embryonic pancreas cells (Fig.S4), demonstrating that cultured ganglia are a suitable source of acetylcholine.

Parasympathetic denervation is traditionally performed surgically by cutting the vagus nerve or chemically by atropine treatment, unfortunately neither of these methods is suitable for denervation of embryonic pancreas, as we don't have surgical access to the developing vagus nerve and atropine specifically blocks muscarinic receptors, but not nicotinic receptors.

Comment 3: It is interesting that adrenergic and cholinergic receptors are expressed in the same MafB+ population based on sequencing, suggesting that they respond to both cholinergic and adrenergic signals. To my knowledge, this is rare. The sequencing studies should be supported by co-localization using immunohistochemistry to show that this endocrine population expresses both cholinergic and adrenergic signaling machinery.

Response: *Islet β cells express adrenergic, muscarinic and nicotinic receptor proteins as the sympathetic nervous system inhibits insulin secretion, while the parasympathetic nervous system promotes insulin secretion (Ganic et al., 2016). We have included immunohistochemical stainings illustrating co-expression of adrenergic and nicotinic receptors with MafB in developing human endocrine cells (Fig. S4).*

© 2023. Published by The Company of Biologists under the terms of the Creative Commons Attribution License (https://creativecommons.org/licenses/by/4.0/).

References:

Artner, I., Le Lay, J., Hang, Y., Elghazi, L., Schisler, J. C., Henderson, E., Sosa-Pineda, B. and Stein, R. (2006). MafB: an activator of the glucagon gene expressed in developing islet alpha- and beta-cells. ***Diabetes*** **55, 297-304.**

Conrad, E., Dai, C., Spaeth, J., Guo, M., Cyphert, H. A., Scoville, D., Carroll, J., Yu, W. M., Goodrich, L. V., Harlan, D. M., et al. (2016). The MAFB transcription factor impacts islet alpha-cell function in rodents and represents a unique signature of primate islet beta-cells. *Am J Physiol Endocrinol Metab* **310**, E91-E102.

Ganic, E., Singh, T., Luan, C., Fadista, J., Johansson, J. K., Cyphert, H. A., Bennet, H., Storm, P., Prost, G., Ahlenius, H., et al. (2016). MafA-Controlled Nicotinic Receptor Expression Is Essential for Insulin Secretion and Is Impaired in Patients with Type 2 Diabetes. *Cell Rep* **14**, 1991-2002.

Mochida, S., Nonomura, Y. and Kobayashi, H. (1994). Analysis of the mechanism for acetylcholine release at the synapse formed between rat sympathetic neurons in culture. *Microsc Res Tech* **29**, 94-102.

Nishimura, W., Kondo, T., Salameh, T., El Khattabi, I., Dodge, R., Bonner-Weir, S. and Sharma, A. (2006). A switch from MafB to MafA expression accompanies differentiation to pancreatic beta-cells. *Dev Biol* **293**, 526-539.

Patterson, P. H. and Chun, L. L. (1977). The induction of acetylcholine synthesis in primary cultures of dissociated rat sympathetic neurons. I. Effects of conditioned medium. *Dev Biol* **56**, 263-280.

Second decision letter

MS ID#: DEVELOP/2022/201009

MS TITLE: MafB-dependent neurotransmitter signaling promotes β cell migration in the developing pancreas

AUTHORS: Sara Bsharat, Emanuela Monni, Tania Singh, Jenny Johansson, Kavya Achanta, Ludivine Bertonnier-Brouty, Anja Schmidt-Crhistensen, Dan Holmberg, Zaal Kokaia, Rashmi Prasad, and Isabella Artner
ARTICLE TYPE: Research Article

I am happy to tell you that your manuscript has been accepted for publication in Development, pending our standard ethics checks. In particular one reviewer suggested clarifying the nomenclature for names, which is important.

Reviewer 1

Advance summary and potential significance to field

The report by Bsharat et al. is a major revision of a prior manuscript by Monni et al. that further explores the role of the transcription factor MafB in pancreatic endocrine cell development and particularly on how MafB regulates the expression of receptors for neurotransmitters and axon guidance genes. In response to prior criticisms, the authors have added significant amount of new data that includes a deeper analysis of ductal and islet morphology, alpha and beta cell area measurements in wild type and MafB mutant pancreata immunohistochemical stainings for TH, vAchT, and MafB in both human and mouse embryonic pancreas, and human scRNA sequencing data. This new data, in addition to an overall editing of the paper to better connect the different parts provides a much more compelling case for MafB being a key driver of neurotransmitter receptor genes in both mouse and human developing endocrine progenitor cells.

© 2023. Published by The Company of Biologists under the terms of the Creative Commons Attribution License (https://creativecommons.org/licenses/by/4.0/).

Comments for the author

The authors should review and improve the nomenclature they use for both mouse proteins and genes. For instance, the murine gene names in the following sentence should be italicized and only the first letters should be upper case. Instead of “Expression of neurotransmitter and axon guidance receptor (ChrnA3, ChrnB4, Robo1 Robo2, P2ry1) genes was predominantly observed…” the correct nomenclature is Chrna3, Chrnb4, Robo1, Robo2, P2ry1 (all in italics). While this issue might be corrected by the journal during page proofing, it would be best if remedied prior to final acceptance.

Reviewer 2

Advance summary and potential significance to field

In this study, Monni et al report a novel role for the MafB transcription factor in islet embryonic development, which involves regulating the neurotransmitter sensing of cells. More specifically, the authors describe how endocrine beta cell migration and clustering depend on nicotinic signaling, a process that might be mediated by islet-nerve cell communication.

Comments for the author

The authors have carefully addressed all my previous concerns. the manuscript is suitable for publication.

Reviewer 3

Advance summary and potential significance to field

The significance of the study lies in defining the role of cholinergic signaling in beta-cell migration, clustering, and islet formation

Comments for the author

The authors have addressed the concerns with additional experiments and textual clarifications that I had in this revised version.

© 2023. Published by The Company of Biologists under the terms of the Creative Commons Attribution License (https://creativecommons.org/licenses/by/4.0/).
